# Ceria-Catalyzed Hydrolytic Cleavage of Sulfonamides

**DOI:** 10.1021/acs.inorgchem.3c04367

**Published:** 2024-01-18

**Authors:** Jiří Henych, Martin Št́astný, Sylvie Kříženecká, Jan Čundrle, Jakub Tolasz, Tereza Dušková, Martin Kormunda, Jakub Ederer, Štěpán Stehlík, Petr Ryšánek, Viktorie Neubertová, Pavel Janoš

**Affiliations:** † Institute of Inorganic Chemistry of the Czech Academy of Sciences, 250 68 Husinec-Řež, Czechia; ‡ Faculty of Environment, Jan Evangelista Purkyně University in Ústí nad Labem, Pasteurova 3632/15, 400 96 Ústí nad Labem, Czechia; § Faculty of Science, Jan Evangelista Purkyně University in Ústí nad Labem, Pasteurova 3632/15, 400 96 Ústí nad Labem, Czechia; ∥ Institute of Physics of the Czech Academy of Sciences, Cukrovarnická 10, 162 00 Prague, Czechia

## Abstract

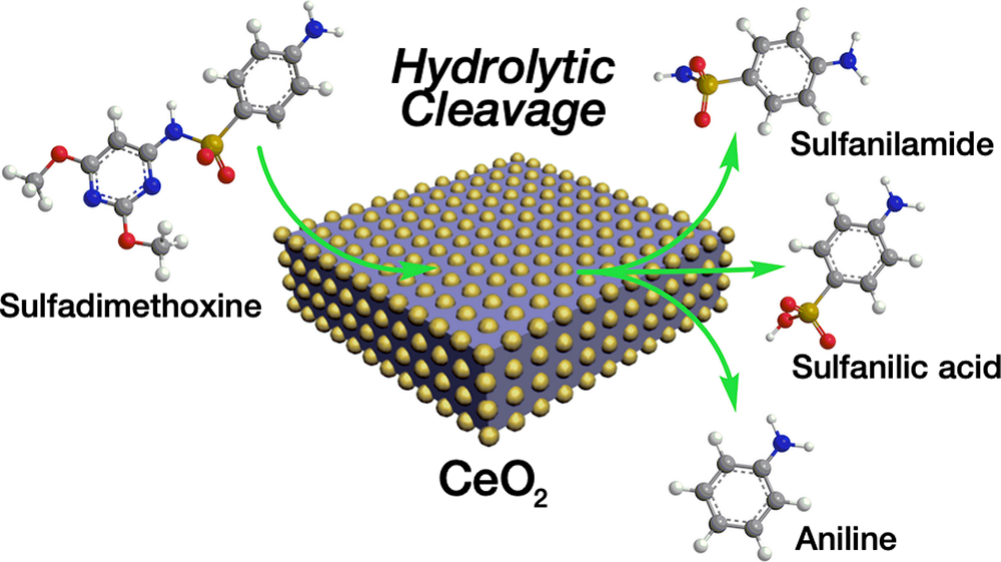

Nanoceria is a promising nanomaterial for the catalytic hydrolysis of a wide variety of substances. In this study, it was experimentally demonstrated for the first time that CeO_2_ nanostructures show extraordinary reactivity toward sulfonamide drugs (sulfadimethoxine, sulfamerazine, and sulfapyridine) in aqueous solution without any illumination, activation, or pH adjustment. Hydrolytic cleavage of various bonds, including S–N, C–N, and C–S, was proposed as the main reaction mechanism and was indicated by the formation of various reaction products, namely, sulfanilic acid, sulfanilamide, and aniline, which were identified by HPLC-DAD, LC-MS/MS, and NMR spectroscopy. The kinetics and efficiency of the ceria-catalyzed hydrolytic cleavage were dependent on the structure of the sulfonamide molecule and physicochemical properties of Nanoceria prepared by three different precipitation methods. However, in general, all three ceria samples were able to cleave SA drugs tested, proving the robust and unique surface reactivity toward these compounds inherent to cerium dioxide. The demonstrated reactivity of CeO_2_ to molecules containing sulfonamide or even sulfonyl (and similar) functional groups may be significant for both heterogeneous catalysis and environmentally important degradation reactions.

## Abstract 2

Ceria nanostructures are able to decompose various sulfonamide antibiotics (e.g., sulfadimethoxine) on their surface by hydrolytic cleavage under ambient conditions to form various degradation products such as sulfanilamide, sulfanilic acid, or aniline.

## Introduction

The sulfonyl group is an important skeletal motif in a number of important industrially produced compounds, especially pharmaceuticals. sulfonamide (SA) drugs belong to the most widely used veterinary antibiotics,^1,2^ but this group also includes, for example, a number of commercial anticancer agents.^3,4^ Heterocyclic compounds bearing the SA group have a privileged role due to their significant and well-known antimicrobial and antiviral effects. It is therefore not surprising that thousands of different SA drugs, analogs and derivatives have been prepared so far.^5^ However, the natural consequence of the use of these substances is their presence in the environment, where they cause serious concern, especially in soil and water.^6^ In addition to directly affecting biota, including animals, they can have a major impact on microbial communities and can also lead to the development of antimicrobial resistance, with a significant impact on human health.

Sulfonamides undergo biotransformation in mammals^1^ to some extent but a significant amount of the parent substance and its metabolites are released and can accumulate within the soil.^7^ In general, SAs are polar compounds, fairly soluble in water, that ionize depending on pH of the medium.^7^ They contain polar functional groups on a nonpolar core, which cause their sensitivity toward acids and bases. However, SA drugs exhibit various physicochemical properties depending on the different side moieties.^1,8^

The most important pathways of natural decomposition of SAs are microbial degradation,^8^ photochemical processes,^9,10^ and hydrolysis.^11^ However, since they are now frequently detected in natural waters, are bioactive, and can induce bacterial resistance, new strategies are being developed to remove poorly degradable SA drugs. Biological, chemical, and/or physical treatment methods include using of bacteria and fungi,^12^ acid hydrolysis, using of strong oxidizers such as chlorine, chlorine dioxide,^13^ ferrate(VI),^14,15^ permanganate in acidic conditions,^16^ or ozone, adsorption on porous adsorbents including activated carbon or zeolites, direct photolysis^10,17^ (also in the presence of H_2_O_2_), semiconductor photocatalysis,^18^ or Fenton-type reactions.^19^ The removal efficiencies and degradation mechanisms of SAs in both chemical and biological degradation systems were reviewed in detail in a recent study.^20^

Transformation of SAs can be also achieved in soil and sediments by abundant natural oxidants, such as manganese oxides,^21^ especially in the presence of humic constituents. Other metal oxides (MOs) with rich redox and acid–base chemistry, occurring naturally in soil and waters or used intentionally as a heterogeneous catalyst, may thus be also capable of initiating or enhancing chemical transformation of SAs. CeO_2_, especially at the nanoscale, is a well-known catalyst that exhibits extraordinary surface reactivity toward various compounds including phosphate esters. The robust and quite unique dephosphorylation activity of Nanoceria toward infamous nerve agents, widespread pesticides, and also highly stable biomolecules with phosphodiester bonds (including DNA) is well documented in the literature.^22−25^

Furthermore, in a recent theoretical DFT study^26^ using acetamide as the main example, the authors proposed that ceria is a potential catalyst not only for phosphate esters but also for the hydrolysis of amides, carboxylates, and amidines among many others that were termed “generalized esters”. Ceria has also previously^27^ been shown to be a highly active, water-tolerant Lewis acid catalyst for hydrolysis reactions in aqueous media.

Inspired by these studies and based on our recent investigation on Nanoceria extraordinary reactivity toward many phosphate esters, we investigated and proved here experimentally for the first time that CeO_2_ is able to chemically transform several SA drugs by hydrolytic cleavage of various bonds, including S–N, C–N, and C–S, as indicated by various reaction products that were identified by HPLC-DAD, LC-MS and NMR spectroscopy. However, of the five sulfonamides tested, three SAs, namely, sulfadimethoxine (SDM), sulfamerazine (SM), and sulfapyridine (SP), were susceptible to cleavage, while the more recalcitrant molecules sulfamethoxazole (SMX) and sulfamethazine (SMZ) were neither adsorbed nor chemically transformed on any of the three used CeO_2_ samples. Nevertheless, the results presented here suggest that the reactivity of CeO_2_ is not limited to well-known dephosphorylation reactions but may also apply to molecules containing sulfonamide or even sulfonyl (and similar) functional groups, which may be significant for both heterogeneous catalysis and environmentally important degradation reactions.

## Materials and Methods

### Sample Synthesis

Nanoceria samples were prepared by three different procedures reported elsewhere^28^ with some modifications. The samples were denoted according to the procedure used as CeAMM, CePER, and CeUREA. Briefly, the CeAMM sample was prepared by precipitation of cerium(III) nitrate aqueous solution with an ammonia solution followed by aging for 4 h at 60 °C in CO_2_-free ambient air without any calcination. The CePER sample was prepared by precipitating an aqueous cerium(III) nitrate solution with sodium hydroxide solution followed by treatment with hydrogen peroxide and refluxing at 100 °C for 24 h without any calcination. The CeUREA sample was synthesized by homogeneous precipitation of aqueous cerium(III) nitrate solution with urea at 90 °C and subsequent calcination at 500 °C/2 h. All prepared samples were dried (or calcined) in atmospheric air and stored in vials under ambient air. See details in the Supporting Information (SI).

### Characterization Methods

Powder X-ray diffraction patterns were collected on a PANalytical X’pert PRO diffractometer equipped with a 1D X′Celerator detector using CuK α radiation (<λ> = 1.5418 Å) in symmetrical Bragg–Brentano configuration in angular range 20–80° 2 theta. The X-ray photoelectron spectroscopy (XPS) apparatus consisting of a SPECS PHOIBOS 100 hemispherical analyzer with a 5-channel detector and a SPECS XR50 achromatic X-ray source equipped with an Al and Mg double anode was used to analyze the surface composition of the samples and the chemical states of the elements (See details in SI). Raman spectra were acquired on a DXR Raman confocal microscope (Thermo Fisher Scientific) using a 532 nm excitation laser. A Thermo Nicolet NEXUS 670 FTIR spectrometer equipped with an MCT detector was used to collect DRIFTS spectra (obtained by accumulating 128 scans with a resolution of 4 cm^–1^) using a gastight Praying Mantis high temperature reaction chamber (Harrick) at room (30 °C) and elevated temperature (200 °C) under the flow of nitrogen gas. The morphology and structure of the particles were studied by transmission electron microscopy (TEM) on the FEI Talos F200X microscope (Thermo Fisher Scientific). Nitrogen physisorption was used to determine the specific surface area (BET method) and porosity (DFT method) of the outgassed (25 °C, 20 h) samples on a Quantachrome Instruments NOVA 3200e (Anton Paar, Austria). Zetasizer Nano (Malvern PANalytical) equipped with a 633 nm helium–neon laser was used for zeta potential measurements as a function of pH with automated titration (the scattering angle 173°; the sample concentration in distilled water between 0.5 and 1 mg/mL). For the quantification of surface hydroxyl groups, acid–base potentiometric titrations on an automatic titrator (794 Basic Titrino, Metrohm, Switzerland) with potentiometric end point determination were used; the detailed measurement procedure was described previously.^29^^1^H and COSY NMR spectra were measured in liquid at 25 °C on a JNM-ECZ400R spectrometer (JEOL Ltd., Tokyo, Japan) in DMSO-*d*_6_. Chemical shifts of ^1^H NMR spectra were referenced to the line of the solvent (DMSO-*d*_6_ δ = 2.49 ppm). NMR spectra were processed with JEOL Delta v 5.3.3 software. The Dionex UltiMate 3000 HPLC-DAD system (Thermo Fisher Scientific, USA) equipped with an autosampler and diode array detector (DAD) and an LC-MS/MS liquid chromatograph 1290 Infinity II system coupled with the 6495A triple quadrupole mass spectrometer (Agilent Technologies, USA) were used for monitoring the hydrolysis reactions. See the measurement details in SI.

### Monitoring Hydrolytic Reactions of SA Drugs

The analytical procedure for monitoring the kinetics of SA drug cleavage was based on a previously^28,30^ developed method for measuring ceria-catalyzed dephosphorylation reactions. Briefly, a powdered sample (50 mg) was weighed in a reagent vial (100 mL) and dispersed in water (49.5 mL) by bath sonication (10 min). The bottle was wrapped in aluminum foil to prevent light, and the reaction was initiated by adding 0.5 mL of stock solution (concentrated at 1 mg/mL) of the selected SA compound. The reagent bottle was placed on a laboratory shaker (at 560 rpm, 3 h, 23 ± 1 °C) and at selected times, 1 mL of suspension was pipetted into an Eppendorf tube (2 mL) and centrifuged (18,000 rpm/2 min), and the supernatants were immediately analyzed by HPLC-DAD. See SI for more details.

### Extraction of Reaction Products

A simple extraction/preconcentration method was developed to identify and quantify reaction products formed by surface chemical reactions that were bound to the Nanoceria surface. The reaction suspension after the adsorption (3 h) was centrifuged (10 000 rpm/5 min), the supernatant was removed, and 2 mL of extraction solution methanol:acetonitrile 1:1 (v/v) was added. The mixture was vortexed, transferred to Eppendorf tube (2 mL), and centrifuged (18 000 rpm/2 min), and the supernatant was collected to 25 mL volumetric flask. The whole procedure was repeated three times, and thus, four extracts (8 mL) were obtained. The volumetric flask was filled to the mark with water, filtered using a syringe filter (NYL, 0.2 μm), and immediately analyzed by HPLC-DAD. The extracts were prepared in the same way at times 5, 30, 60, 90, and 180 min. Blank experiments were also carried out without a catalyst to exclude spontaneous decomposition of SA in various solvents, adsorption on glass, adsorption on a lid, and so forth. Solvent extracts were diluted 100× for LC-MS/MS analysis.

For NMR spectroscopy, the direct analysis of the reaction solution of ceria with SDM was performed. However, due to the low sensitivity of NMR, the starting concentration of SDM in the reaction solution was 1, and 100 mg/mL ceria was used.

## Results and Discussion

### Materials Characterization

**Figure 1:**
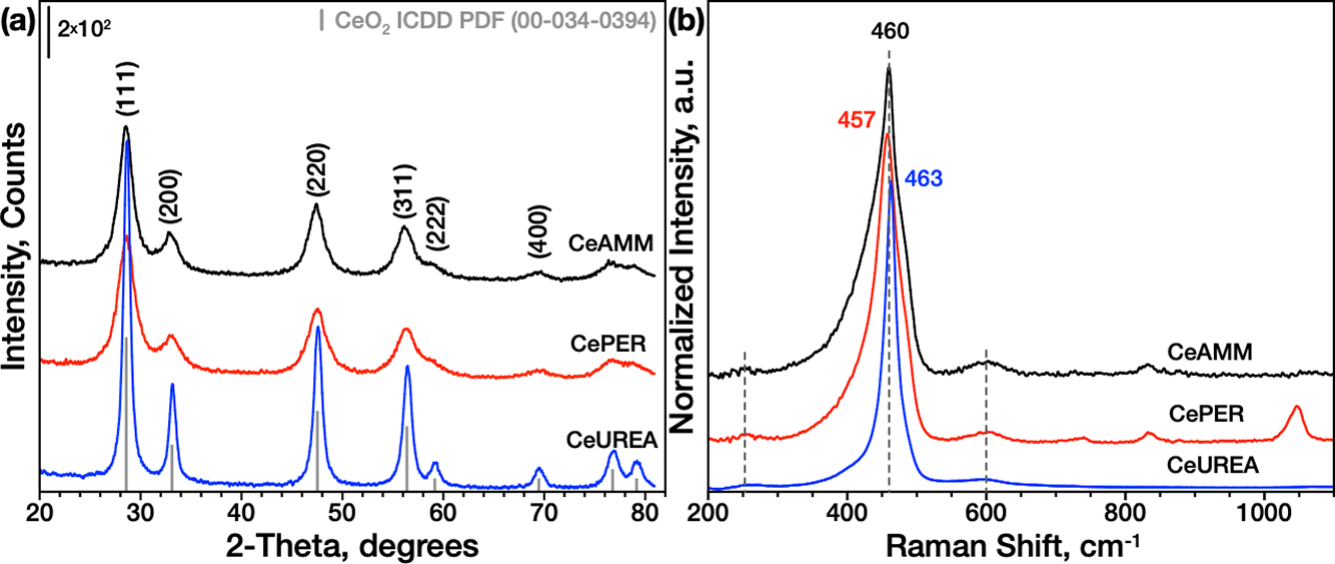
(a) X-ray diffractograms and (b) Raman spectra of the prepared ceria samples.

The three Nanoceria samples prepared by the selected water-based precipitation methods differ significantly in particle size and morphology, surface area and porosity, concentration of defects, and surface properties as discussed in our previous work.^28^ Some additional properties that are relevant to the surface properties and the studied cleavage reactions of SAs were also investigated here. XRD (Figure 1a) confirmed the cubic fluorite ceria crystalline structure in all three samples; the Sherrer equation was used to calculate the average crystallite size (Table 1), which was 4.7 nm for both CeAMM and CePER samples and 11.1 nm for CeUREA, respectively. However, from the calculation of crystallite sizes in different directions (Table S1), it is evident that the CeAMM sample has almost uniform crystallite size in all directions, while the CePER sample shows more irregular particles with elongated (111) planes.

**Table 1:** Crystallite Size, Specific Surface Area (SA), Pore (and Micropore) Volume, the Number of Surface Hydroxyl Groups per Weight (*q*_OH_), and the Relative Oxygen Vacancy Concentration (Calculated from Raman Spectra as *I*_*600*_*/I*_*460*_) of Prepared Ceria Samples

sample	cryst. size (XRD), nm	SA (BET), m^2^/g	pore volume (DFT), cm^3^/g	micropore area, m^2^/g	micropore volume, cm^3^/g	*q*_OH_, mmol/g	*I*_*600*_*/I*_*460*_
CeAMM	4.7	142.9 ± 0.3	0.400 ± 0.009	13.4 ± 0.8	0.005 ± 0.001	0.109	0.040
CePER	4.7	192.2 ± 0.7	0.117 ± 0.001	8.5 ± 0.9	0.003 ± 0.001	0.185	0.030
CeUREA	11.1	51.0 ± 1.2	0.050 ± 0.001	0	0	0.130	0.022

Raman spectra consistent with XRD results show the main CeO_2_ symmetric breathing vibration^31^ of oxygen atoms around each Ce^4+^ cation (F_2g_), which was centered at 463 cm^–1^ for CeUREA, while it was red-shifted to 460 cm^–1^ for CeAMM and 457 cm^–1^ for CePER. Generally, the red-shift and asymmetrical broadening of F_2g_ are caused by decreasing particle size and any lattice distortion.^31^ As CeAMM and CePER samples have similar crystallize sizes, the larger F_2g_ peak red-shift of the latter may indicate a larger number of defects. All samples show asymmetrical peak fronting, which is more visible for CeAMM and CePER samples due to the larger broadening caused by their smaller particle size. The band at ca. 250 cm^–1^ can be associated with the surface termination of clean (111) surfaces due to the defects and surface OH groups,^31^ while the band at 600 cm^–1^ is often attributed to Frenkel-type oxygen defects.^32^ Both bands are visible in spectra of all samples but are more pronounced in CeAMM and CePER samples, which might be also related to their smaller particle size. A band at 830 cm^–1^ recognized also only on these two samples was assigned to peroxides, which can form upon adsorption of oxygen onto two electron defects.^33^ In CePER, two additional bands at 735 and 1041 cm^–1^ belong to residual NO_3_^–^ species from the synthesis. The integral intensity ratio of the bands at ∼600 (*I*_*600*_) and∼460 (*I*_*600*_) can be used to quantify the relative oxygen vacancy concentration (see Table 1). As evident from the data, the O-vacancy concentration decreases in the order CeAMM > CePER > CeUREA.

**Figure 2:**
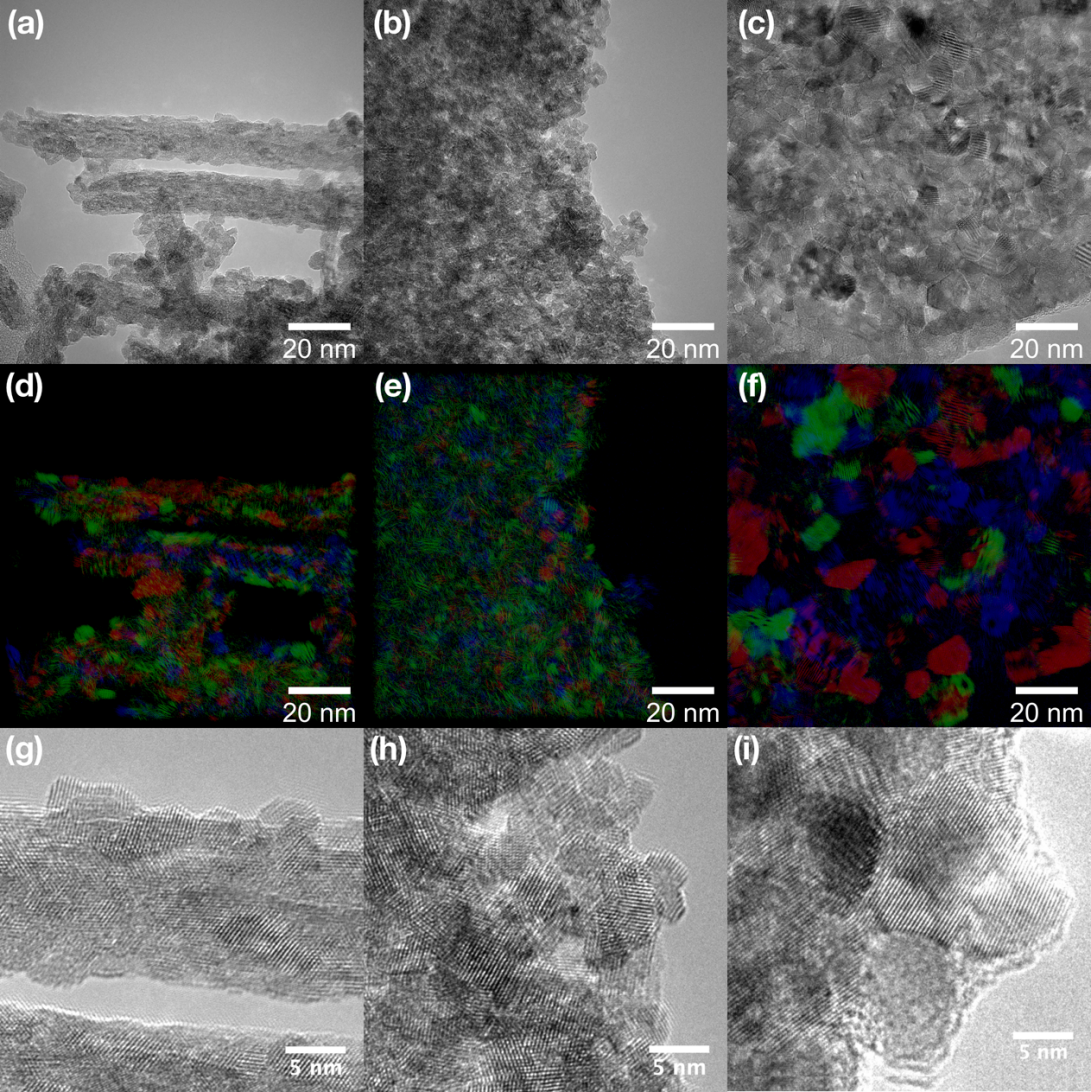
TEM images of (a) CeAMM, (b) CePER, (c) CeUREA samples, (d–f) color FFT visualization of the exposed planes in the same samples, and (g–i) HRTEM images of the same samples.

The specific surface area of samples obtained by isothermal nitrogen physisorption (Table 1) was relatively high for CePER (192.2 m^2^/g) and CeAMM (142.9 m^2^/g) samples consistent with their small crystallites, while it was 51.0 m^2^/g for the CeUREA sample. Interestingly, the shape of the isotherm (Figure S1a) was very similar for CePER and CeUREA samples showing type H4 hysteresis loop,^34^ while the CeAMM sample exhibited type H3. This indicates a similar interparticle pore structure for CePER and CeUREA that are both formed by dense aggregates, while the H3 loop in CeAMM suggests nonrigid aggregates or some macropores that are not completely filled,^34^ which is consistent with TEM analysis (Figures 2 and S2). The very different interparticle pore structures may explain the different surface areas of CePER and CeAMM samples despite their similar average crystallite size. This is also evident from the different pore size distribution (Figure S1b), which reveals mesopores in CePER and CeUREA samples with the maximum at around 20–30 nm, while larger interparticle pores (with the maximum at 50–70 nm) were identified in the CeAMM sample. CePER and CeAMM samples also contain a noticeable number of micropores (pores below 2 nm).

The morphology of the prepared ceria nanostructures is very different, as shown by TEM analysis (Figures 2 and S2). While the CeAMM sample contained loosely aggregated rod-like particles, the CePER sample formed dense irregular aggregates and the CeUREA samples consist of microplatelets. Nevertheless, in all three samples, these secondary structures are formed by significantly smaller highly crystalline aggregated primary nanoparticles, as documented by HRTEM (Figure 2) and XRD. The observed particle sizes are fairly consistent with XRD data, showing polyhedral and angular particles with sizes up to 5 nm in the case of CeAMM and CePER samples, respectively. The CeUREA sample consists mainly of rounded larger particles with irregular size (between 5 and 15 nm). The random orientation of particles exposing various crystal planes and different particle sizes between samples can also be visualized with different colors using FFT (Figure 2).

Differences in particle size, morphology, and aggregation (resulting in different interparticle porous structures) together with sample treatment temperature significantly affect which crystal planes are exposed, along with defect structure, reducibility, surface chemistry including hydroxyl groups, and interactions of Nanoceria with water.^35^ All of these aspects have a crucial influence on the reactivity and adsorption properties of Nanoceria.

XPS analysis (Table 2, Figure S3) confirmed the expected surface elemental composition (Ce, O) without any impurities in all three samples. Nitrogen from residual nitrates was also not identified in the CePER sample (in contrast to Raman spectra) probably because nitrates are not preferably located on the surface. In all three samples, the O/Ce ratio is highly overstoichiometric, which may be caused by their preparation in water in ambient air facilitating the formation of oxygen-containing surface groups and also the abundance of water molecules associated with their surface. The formation of surface −OH groups that are preferentially formed on defects by dissociation of water^36^ may also indicate high reactivity of the prepared nanoparticles. Also, the contribution of carbonates/carboxylates that are commonly formed by CO_2_ dissociative adsorption on ceria stored at ambient air^37^ should not be neglected, together with the presence of the cerium-oxo hydroxide phase, which is typical for microporous ceria structures prepared in water without calcination.^38^

**Table 2:** Surface Elemental Composition of Ceria Samples Obtained by XPS

sample	Ce, atom %	O, atom %	O/Ce	Ce^4+^/Ce^3+^	–OH, atom %
CeAMM	25.2	74.8	3.0	8.7	3.4
CePER	21.1	78.9	3.7	3.1	12.8
CeUREA	25.7	74.3	2.9	6.4	5.0

Interestingly, CeAMM and CeUREA samples have a practically identical O/Ce ratio of ∼3.0 despite the fact that CeAMM was prepared at 60 °C without any calcination, while CeUREA synthesized at 90 °C was further treated at 500 °C; that is, it was significantly more dehydrated. However, as shown by XPS, CeUREA had a higher concentration of both Ce^3+^ and −OH groups compared to CeAMM. Note that in all three samples, the number of Ce^3+^ and −OH groups is correlated; that is, the higher the number of Ce^3+^, the higher the number of −OH groups, which supports the assumption^36^ that water dissociation occurs more easily on surface defects. This indicates that CeUREA has slightly more defects (and thus Ce^3+^ and −OH groups) compared to CeAMM. The latter, on the other hand, has significantly smaller particles that are less aggregated and thus contain more edges, corners, and surfaces that can have more exposed surface oxygen atoms (and oxygen-containing surface groups), which consequently leads to a similar O/Ce ratio of these two samples.

The CePER sample has an even higher O/Ce ratio (3.7) due to both the small particle size and the large number of defects and thus reduced Ce^3+^ sites (2.8 and 2 times larger compared to CeAMM and CeUREA) and surface −OH groups consistent with Raman spectra. As it was elaborated in our previous work,^28^ higher defect concentration in CePER is most likely due to hydrogen peroxide used in the synthesis, which has oxidation–reduction properties and also significantly affects the nucleation and crystallization process.

It is worth noting that the relative number of OH groups found by XPS is well correlated with the number of OH groups obtained by acid–base titration (calculated from the two equivalence points on the titration curve as previously^29^ described) shown in Table 1; that is, the number of −OH groups decreased in the order CePER > CeUREA > CeAMM.

In situ DRIFT spectra (Figure S4) were measured at room temperature (25 °C, dotted lines) and 200 °C (solid lines) under a flow of nitrogen in order to study the structure of surface species such as −OH groups. The acid properties of the hydroxylated surface are more effective, and surface hydroxyl group centers also allow reorganization of the adjacent water molecules maximizing the number of H-bonds both to the surface and in between the H_2_O molecules in subsequent layers. As a consequence, improved wetting might appear, which also will affect the reactivity and surface chemical reactions.^39^

As expected, the broad band between 2500 and 3500 cm^–1^ together with the associated band at 1635 cm^–1^ of physisorbed water decreased significantly upon heating at 200 °C and the isolated OH groups become visible in the spectra. Bands at 3701, 3651 (with shoulder at 3627), and 3531 cm^–1^ were assigned in the CeAMM sample to terminal (type I), bridging (type II), and hydroxide-like OH, respectively.^35,38,40,41^ The broad band at 3531 cm^–1^ may also contain the contribution of triply bridging OH groups (type III). In the case of isolated OH groups, the higher the wavenumber, the more basic the OH groups. While both terminal (more basic) and bridging (more acidic) hydroxyl groups were distinguished in CeAMM and CeUREA samples, although in the latter with a significantly lower intensity, in the CePER sample, mainly bridging (acidic) OH groups were identified. The acidic OH groups that are characterized by higher proton mobility^40^ can easily protonate basic molecules, and the proton can be exchanged during catalytic reactions. In CeAMM and CePER samples, the band at 3531 cm^–1^ belonging to the cerium-oxo hydroxide phase is evident, which is more abundant in the latter and is typical for microporous ceria structures prepared in water without calcination.^38^ This may also explain the high overstoichiometric O/Ce ratio of CePER found by XPS.

**Figure 3:**
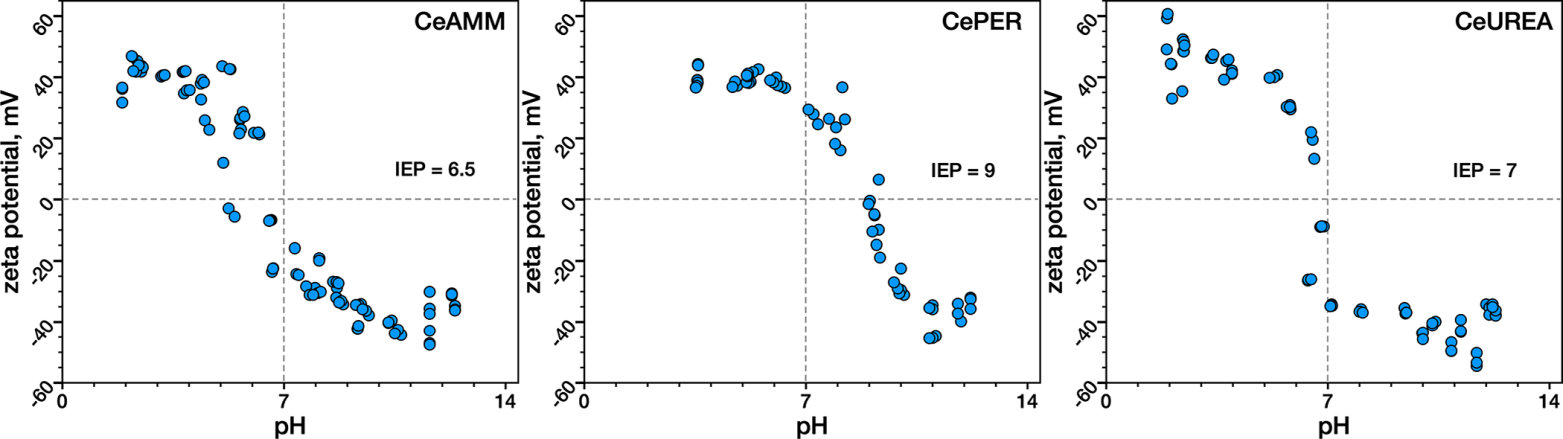
Zeta potential measurements of prepared Nanoceria samples as a function of pH.

The zeta potential of ceria samples as a function of pH was measured to evaluate the surface properties and colloidal stability of the samples (Figure 3). As evident, all three samples have high values of zeta potential around +40 mV at low pH (2–4) as a result of adsorption of H_3_O^+^ ions and highly negative zeta potential (−40 mV) at pH 10–12 due to surface adsorption of OH^–^ ions. However, the samples differ significantly in the isoelectric point (IEP) in which the particles carry no net charge. While CeAMM has an IEP of around 6.5 and CeUREA around 7, the IEP of the CePER sample is around 9, which explains its significantly higher colloidal stability at pH 5–7, at which the other two samples already lose their colloidal stability. This indicates the very different surface properties of CePER as well as its higher buffering ability at pH below 7, which is likely related to the high oxygen defect concentration and large number of surface −OH groups in this sample, consistent with XPS and Raman spectroscopy investigations. The hydrodynamic particle size in solution as a function of pH for each sample also obtained by DLS measurements is shown in Figure S5.

### Reactivity of Ceria toward SA Drugs

**Figure 4:**
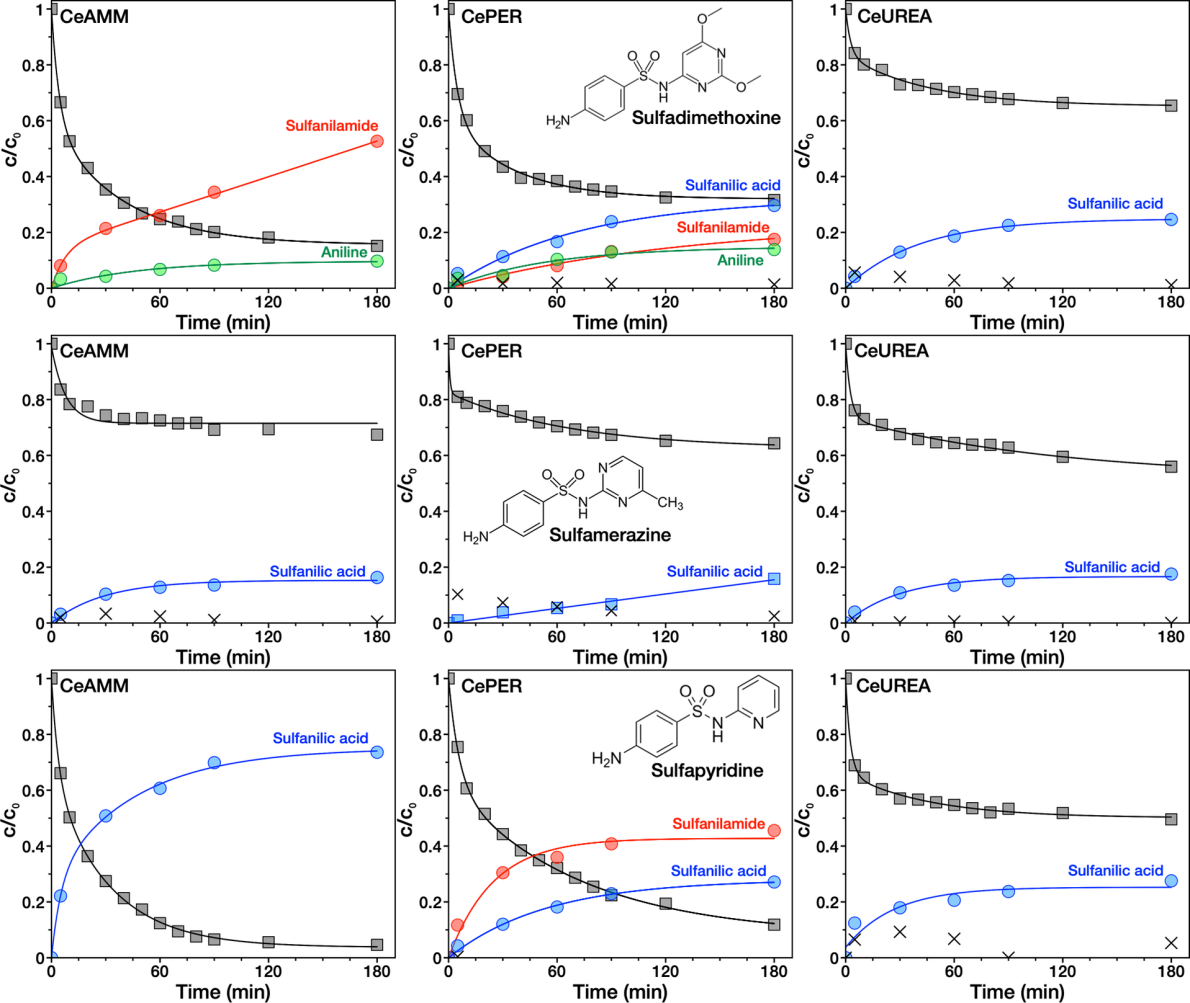
Kinetics of ceria-catalyzed cleavage of SDM (top), SM (middle), and SP (bottom) and formation of various reaction products using different Nanoceria samples in water. Color-coding: Black squares—residual concentration of SA drug in solution (before extraction); red (sulfanilamide), blue (sulfanilic acid), green (aniline), and black crosses (unreacted SA drug extracted from the sample surface)—relative concentration of residuals obtained after extraction.

Nanoceria samples were used for adsorption and cleavage of SDM, SM, and SP in aqueous solution without any illumination, activation, or pH adjustment (Figure 4). As shown by the kinetic curves (black squares) in Figure 4, all three antibiotics were gradually removed from the aqueous solution by all three ceria samples, indicating that Nanoceria can act as an efficient adsorbent for certain SA drugs. In general, the removal efficiency decreased in the order SP < SDM < SM for all three samples. It must also be mentioned here that the adsorption of other SAs, namely, sulfamethoxazole and sulfamethazine, was also tested. Neither of these drugs was almost at all adsorbed on any of the ceria samples (data not shown). This suggests that the structure of the SA drug, the attached moieties, and probably their polarity have the main influence on drug adsorption on the CeO_2_ surface. Also, in comparison to other hydrophobic organic chemicals, SMX adsorption is relatively more complicated because it may exist as three types of species at different pH.^42^ Furthermore, in the removal of SMX (and SMZ), the main mechanisms for effective adsorption are hydrophobic interaction, hydrogen bonding (H-bonding), and π–π electron donor–acceptor interaction,^43^ which favors the large specific surface area and surface functional groups of carbon materials over the characteristics of CeO_2_ (or other metal oxides). The most efficient sample for the adsorption of all three SAs was the CeAMM sample, but it was very similar to the CePER sample. CeUREA was the least effective, but it showed similar adsorption kinetics for all three SA drugs, which indicates its lower specificity to the different substrates.

### Extraction and Identification of Reaction Products

Although the formation of degradation products in the reaction solution was not observed, to investigate possible chemical transformation of adsorbed SAs, solvent extraction of all samples was performed at selected times. Interestingly, several reaction products were found in extracts of all samples, and they were identified by comparing the HPLC retention times of the products with the standards of substances that were selected as possible reaction products according to previous studies.^11,15^

Three major reaction products, sulfanilic acid, sulfanilamide, and aniline, were identified, and their kinetics are shown by the red, blue, and green curves in Figure 4, respectively. Extracted unreacted SA (if found) is shown by crosses in the graphs. The identified reaction products are identical to those found by Białk-Bielińska et al.^11^ in acid hydrolysis (after days and without any solid catalyst) of several SAs in solution suggesting that hydrolysis (but ceria-catalyzed) is the most likely reaction mechanism in this study. sulfanilic acid is the most commonly observed reaction product, followed by sulfanilamide and aniline. As evident, the products formed and their kinetics are different on each ceria sample, but in general, all three samples are capable of cleaving the SA drugs tested, proving robust reactivity toward SAs inherent nature to cerium dioxide.

CeAMM is the most efficient sample, which may be due to its large surface area and suitable morphology and porosity. CePER has a similarly high efficiency; thanks to the largest surface area and a large number of surface hydroxyl groups. Due to the high defect concentration, it also has the ability to cleave different bonds in SAs and generate various reaction products (discussed below). However, the resulting products may also be more tightly bound to the surface, which may inhibit its overall reaction efficiency. Even CeUREA showed relatively good reactivity despite its almost four times smaller specific surface area (compared to CePER) possibly also due to the relatively large number of surface −OH groups.

**Figure 5:**
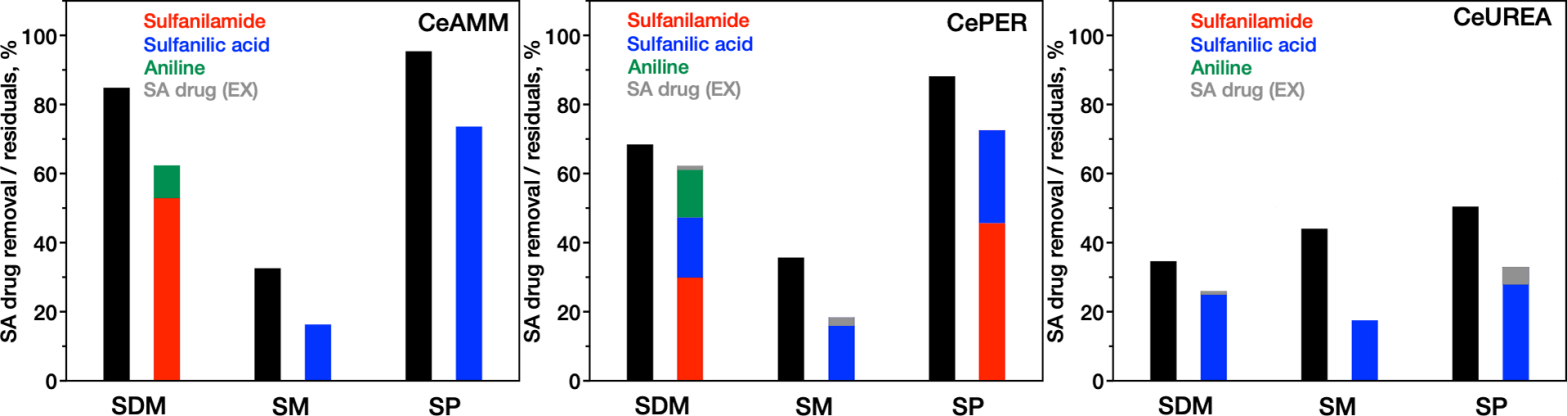
Removal efficiency of different SA drugs (black columns) and distribution of products formed (and extracted unreacted SA drugs) by the ceria-catalyzed hydrolysis of SAs in water.

The removal efficiency and composition of reaction products on each sample are presented in Figure 5. As can be seen, the CePER sample shows the greatest variation of the formed products, while only sulfanilic acid was identified in reaction of CeUREA with all three SAs. This is probably related to the variability and strength of the possible active sites on the surface of different ceria samples as well as to the susceptibility of individual bonds in SAs to their cleavage (as will be discussed below). It is obvious from Figure 5 that the amount of products formed was not completely stoichiometric with respect to the decomposed drug. The difference in this ratio was highest for SM but was observed systemically for all samples and drugs. Although not completely clear, this may be due to imperfect extraction process and strong adsorption of products or unreacted drug on ceria.

### Effect of pH on the Reaction

**Figure 6:**
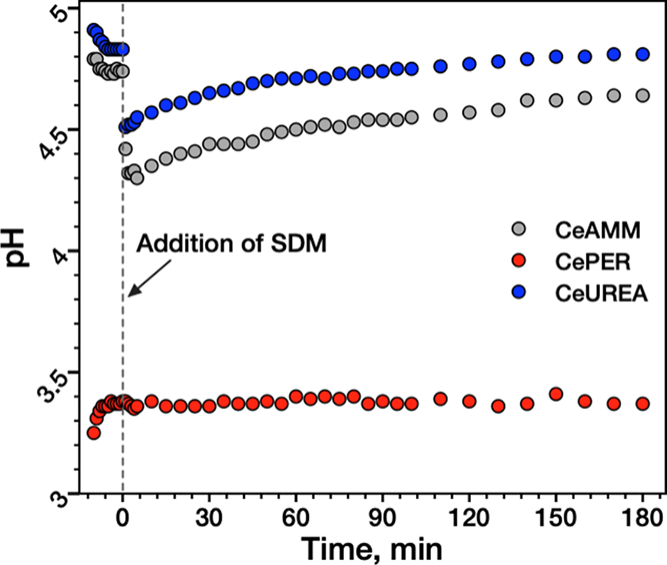
PH measurements before and during the degradation of SDM in the presence of Nanoceria in RO water.

Significant changes in pH were observed during the adsorption and reaction of SDM as a representative compound (Figure 6). Dispersing ceria powder in reverse osmosis (RO) water resulted in a decrease in pH from ∼6.6 to 4.83, 4.74, and 3.38 for CeUREA, CeAMM, and CePER, respectively, likely due to the dissociation of water on the ceria surface and the release of protons into the solution. The addition of SDM to the reaction mixture caused a further rapid decrease in pH to 4.30 and 4.51 for CeUREA and CeAMM, respectively, followed by a slow gradual increase in pH during the reaction. Note that the addition of SDM alone to pure RO water resulted in a decrease in pH from 6.61 to 6.30 (Figure S6). The gradual increase in pH in the course of the reaction is likely related to the formation of products, where protons from the solution are used to regenerate surface acidic OH groups on Nanoceria that are consumed during the reaction. Alternatively, some protons may be consumed directly in the process of forming products.

In contrast to CeAMM and CeUREA, for the CePER sample, there was virtually no change in the pH during the reaction. This suggests, together with the zeta potential measurements, a much higher buffering capacity, probably due to a higher amount of acidic OH groups on its surface (see XPS). The pH changes induced by the addition of SDM to the reaction mixture and its hydrolysis are probably too small compared to the amount of H_3_O^+^ ions released into the solution by water dissociation on the CePER surface.

Since ceria addition caused a significant decrease in the pH of the reaction solutions, the effect of the acidic pH alone on possible SA drug hydrolysis was evaluated. Thus, two reaction solutions without ceria but with pH adjusted (by hydrochloric acid) to pH 3.38 and 4.79 were prepared, and spontaneous decomposition of SDM was studied (Figure S7). As is evident, no significant degradation of SDM was observed at pH 4.79. At pH 3.38, the evaluation of the chromatogram was more complicated with SDM forming a double peak probably due to the presence of both neutral and protonated forms of SDM (data not shown); but importantly, there was no significant decrease in SDM concentration over the time period measured. This indicates that the SDM hydrolysis is indeed due to the unique surface functionalities of ceria and not due to a change in the pH of the reaction solution.

### Influence of Solvent Used

SN_2_ nucleophilic substitution has been proposed previously^30,44^ as the main mechanism of dephosphorylation reactions on Nanoceria. In addition to nucleophile and the leaving group, the solvent has an important effect on the reaction rate.^45^ The dephosphorylation is promoted in polar aprotic solvents, while it is hindered in polar protic solvents due to the solvation effect and interaction of the nucleophile with the solvent. Therefore, to investigate whether a similar reaction mechanism might be involved in the ceria-catalyzed hydrolysis of SAs, we tested how the reaction would proceed in acetonitrile (Figure S8) instead of water, which generally accelerates ceria-catalyzed dephosphorylation, as found previously.^28,46^

Surprisingly, acetonitrile (ACN) had a generally detrimental effect on the reaction rate, albeit slightly different for each sample. While for CeAMM, only a slight decrease of the SDM decomposition rate and an improved rate for sulfanilamide formation was observed; both the SDM hydrolysis rate and product formation rate were substantially hindered for the CePER sample. The reaction rate almost did not change for the CeUREA sample. Since hydrolysis by surface-bound OH groups on ceria surfaces appears to be the main reaction mechanism (similar to previously studied^30^ dephosphorylation reactions), it can be assumed that a significant amount of surface −OH groups is consumed during the reaction and their availability may thus be a crucial factor. Therefore, the observed higher reaction rate in water may be related to sufficient regeneration of consumed OH groups that cannot proceed in ACN. This effect might be more important than the solvation effects on nucleophilic OH groups and hydrogen bonding of associated water molecules.

For CeAMM, the overall good reaction rate in ACN may also be related to the improved desorption of sulfanilamide. CeUREA shows practically identical reaction rates in both solvents proving its robustness compared to other ceria samples, which was also observed previously^28^ in the case of dephosphorylation reactions.

### LC-MS/MS Analysis

Due to the better sensitivity and accurate determination of the reaction products, the LC-MS/MS method was further used. Figures S9 and S10 show the basic molecular ion signals of the compounds used as reference standards, namely, SDM (*m*/*z* 311[M + H]^+^), SM (*m*/*z* 265[M + H]^+^), SP (*m*/*z* 250[M + H]^+^), and expected reaction products sulfanilic acid (*m*/*z* 174[M + H]^+^), sulfanilamide (*m*/*z* 173[M + H]^+^), and aniline (*m*/*z* 94[M + H]^+^) obtained via positive ion electrospray ionization (ESI+) mass spectrometry. Although the separation of low-molecular-weight degradation products with similar *m*/*z* values (such as sulfanilic acid and sulfanilamide) can be problematic, their chromatographic separation allows for more accurate resolution. In addition, note that the separation of aniline and its ionization under the stated conditions can be problematic due to impaired ionization and very short retention time, which may cause interference with the injection peak.

**Figure 7:**
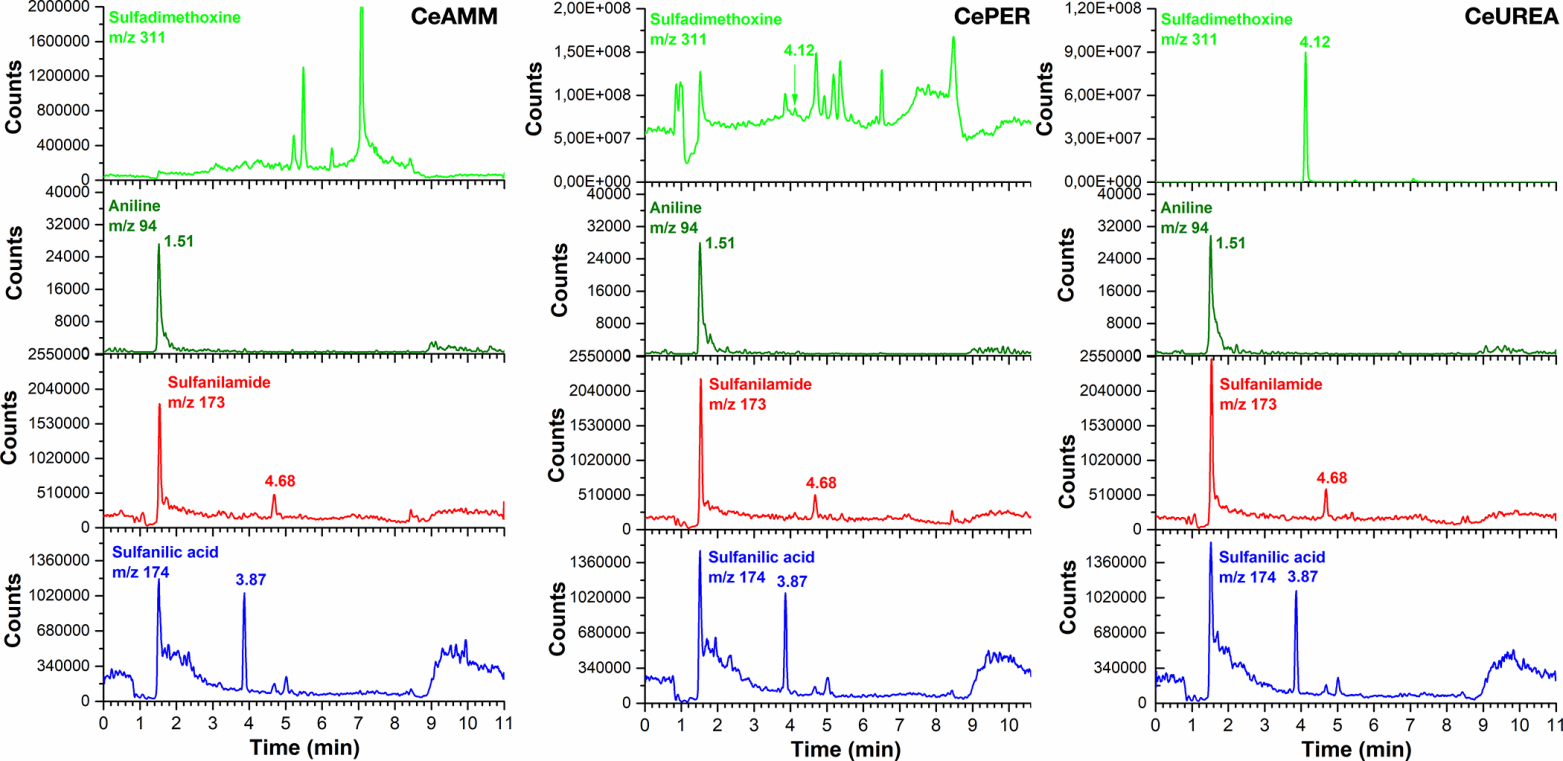
LC-MS analysis of representative samples obtained by extraction of different ceria samples after the reaction with SDM.

A representative analysis of all three ceria sample extracts obtained after 180 min of reaction with SDM is shown in Figure 7. Analysis of all three ceria samples with SM and SP is shown in SI in Figures S11 and S12. Note that the total ion chromatograms of the SA parent drug (SDM) mixed with different ceria samples (in Figure 7) are different, indicating the high reactivity of ceria and the immediate formation of degradation products. The mass spectra of the separated products in Figure 7 were matched to reference standards, thus confirming the formation of the expected reaction products. As can be seen, the three products, sulfanilic acid, sulfanilamide, and aniline, were identified in all extraction solutions. Note that due to the significantly higher sensitivity of MS, all three products were also found in extracts where the HPLC-DAD method was unable to determine them. More importantly, this method provides further evidence that SA drugs undergo hydrolysis in the presence of CeO_2_ to form products previously recognized by HPLC-DAD.

### ^1^H NMR Spectroscopy Analysis

**Figure 8:**
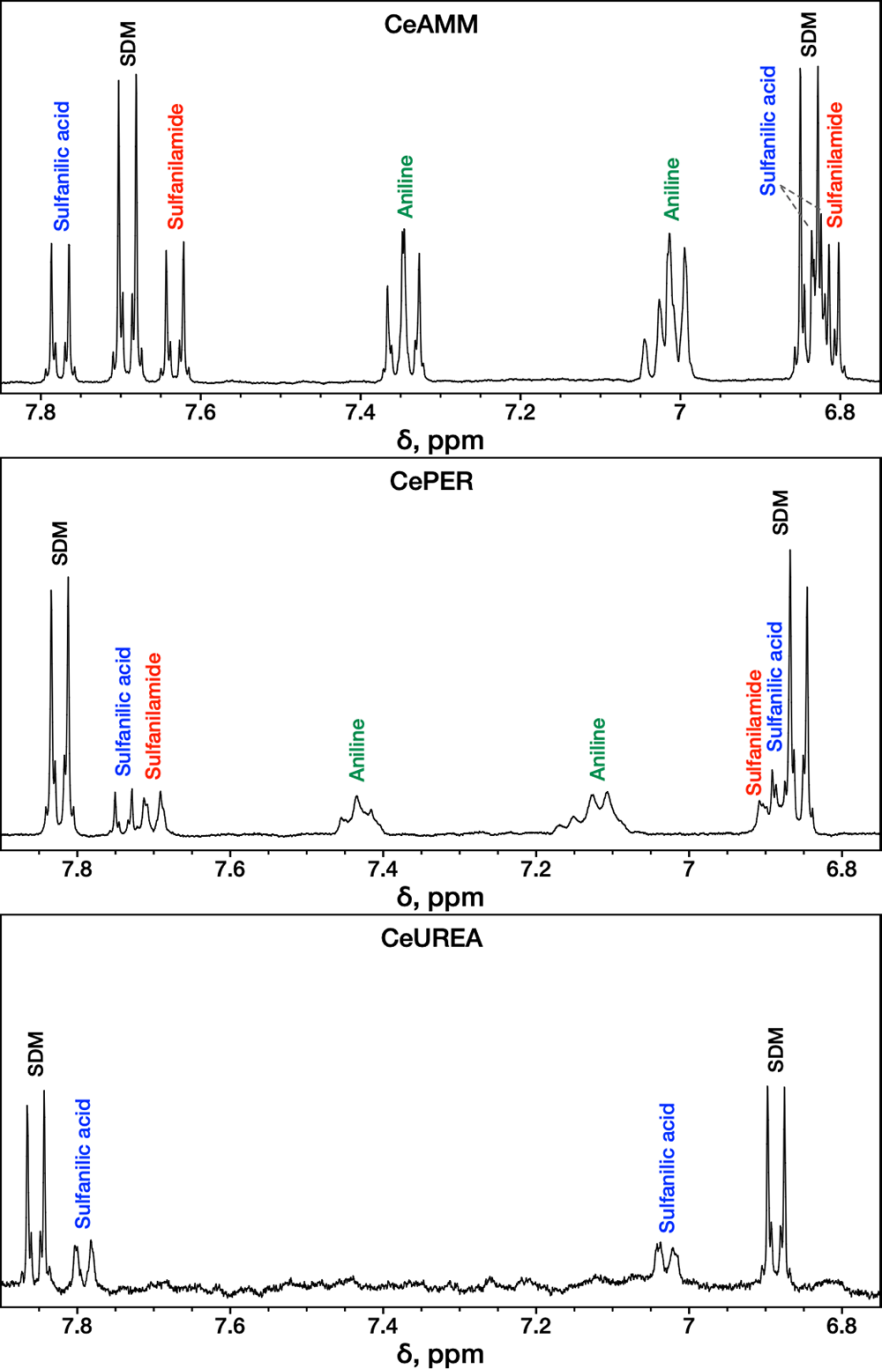
^1^H NMR spectra of the reaction solutions of the prepared ceria samples with SDM after 180 min of reaction and centrifugation.

^1^H NMR spectroscopy was employed to further confirm the formation of hydrolysis reaction products by a fundamentally different method. The reaction suspensions of all three ceria samples with SDM after 180 min of reaction were analyzed. The spectra are presented in Figure 8, and the complete ^1^H–^1^H COSY spectra can be found in the SI (Figures S13–S15) along with the spectra of the individual expected compounds (Figures S16–S19) that were used as references.

Signals belonging to the SDM were observed in the spectra of all three samples. These are the signals with the highest intensity belonging to protons of CH groups, protons of the NH_2_ group, and the proton of the pyrimidinyl ring. For CeAMM, the signals of SDM are at 7.69 ppm, 6.84 ppm (2 × app d, AA′XX′, ^3^*J* = 8.7 Hz, ^3^*J* = 8.8 Hz, 2 × 2H, 4 × CH), 6.13 ppm (s, 1H, CH), and 6.29 ppm (br s, 2H, NH_2_). The signals of the degradation products aniline, sulfanilic acid, and sulfanilamide were also identified in the spectra of CeAMM and CePER samples. The CH groups of aniline were found as multiplets at approximately 7.35 ppm (2H) and 7.01 ppm (3H) for the CeAMM sample and at 7.43 ppm (2H) and 7.14 ppm (3H) for CePER.

However, doubtless assignment of sulfanilamide and sulfanilic acid solely on the basis of the NMR data was not possible, given their similar appearances, identical coupling constants, and integration. Nevertheless, the presence of both decomposition products could be confirmed. Part of the signals belonging to sulfanilic acid and sulfanilamide were found around 7.78 and 7.63 ppm (CeAMM) and 7.74 and 7.70 ppm (CePER). These signals have a characteristic coupling constant (^3^*J* = 9 Hz) and proton count (2H, 2 × CH) according to the individual spectra of these compounds. Moreover, through the ^1^H–^1^H COSY experiment, the signals were shown to correspond to signals concealed within the multiplets at 6.82 (CeAMM) and 6.87 ppm (CePER). However, the signals were only tentatively assigned to sulfanilic acid and sulfanilamide, respectively, in Figure 8.

Only signals for SDM and one product were observed in the CeUREA sample spectrum, which is in good agreement with the HPLC-DAD results and the fact that this sample had the lowest activity. Although the product cannot be without a doubt assigned to sulfanilamide or sulfanilic acid, based on the HPLC-DAD, we assume that the signals belong to sulfanilic acid. What is important, however, is that the formation of several hydrolysis products was undoubtedly confirmed in all three reaction mixtures with the help of NMR spectroscopy.

### Reaction Mechanism

**Scheme 1:**
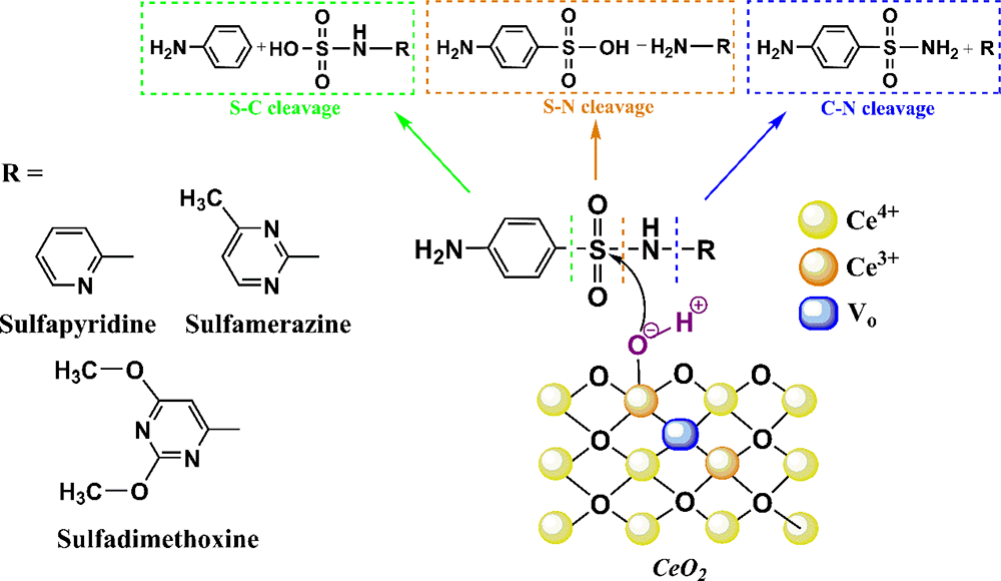
Possible Degradation Mechanism of Cerium-Catalyzed Hydrolysis of the Selected SA Drugs

Białk-Bielińska et al.^11^ described in detail the hydrolysis of SA drugs under different environmental conditions (pH and temperature). Sulfanilamide, sulfanilic acid, and aniline were identified as the main reaction products of sulfisoxazole, sulfadimethoxine, sulfamethoxypyridazine, and sulfachloropyridazine. However, the hydrolysis was carried out at low pH (4.0), at temperatures between 20 and 70 °C, and the reaction rate was very slow, with the highest conversion of only 41% for sulfachloropyridazine after >30 days (at 70 °C). The reaction products were identified using the HPLC-UV method based on a comparison of retention times with the standards. Prior studies^10,17^ on the photolysis of SAs also showed aniline, sulfanilamide, and sulfanilic acid as the photodecomposition products. Identical products were also found in this work by HPLC-DAD, LC-MS/MS, and NMR spectroscopy methods, which indicates that ceria-catalyzed hydrolysis is the likely reaction mechanism. However, the catalytic reaction on CeO_2_ is significantly more effective, as a higher conversion was achieved in a matter of minutes at room temperature and without pH adjustment. Based on these results, a tentative mechanism of ceria-catalyzed SA drug hydrolysis is proposed in Scheme 1.

#### S–N Bond Cleavage

One of the most common reaction pathways of SA drugs is cleavage of the sulfonamide (S–N) bond by nucleophilic sulfonyl substitution on the sulfonamide S atom, which yields sulfanilic acid and the corresponding H_2_N-*R* substituent.^11,47^ sulfanilic acid was found as the main reaction product in this study. Kim et al.^15^ proposed that the S–N bond cleavage is favorable under acidic conditions, as the protonated amine group tends to become less reactive toward nucleophiles, while the sulfonic group becomes the most reactive site. Moreover, under acidic conditions, the leaving group is neutral (while under basic conditions, the eliminated amino-heterocycles are negatively charged), which facilitates its substitution.^11^ The surface metal cations acting as Lewis acidic sites together with oxygen-containing species (such as −OH groups acting as nucleophiles) on the CeO_2_ surface probably can promote the hydrolysis of SAs through a sulfonyl group substitution mechanism (Scheme 1). While Ce^4+^ with a significant electron-withdrawing ability can activate the substrate^30^ (Lewis acid activation), the OH group attached to the Ce^3+^ cation represents a very effective nucleophilic agent (compared to the Ce^4+^-bound OH group). Another important effect may be water dissociation on highly defective CeO_2_ to form surface-bound – OH groups and H_3_O^+^ ions that can be released into the solution and thereby lower the pH, which can further promote hydrolysis.

#### C–N Bond Cleavage

The C–N bond cleavage is another pathway of SA hydrolysis forming sulfanilamide–the main unit of sulfonamides. The cleavage may occur via an aromatic nucleophilic substitution mechanism in the Heterocyclic aromatic ring (Heterocyclic rings, especially six-membered, have a strong electron-withdrawing ability that favors nucleophilic attack).^11^ The C–N cleavage with forming sulfanilamide was also commonly observed during the photodegradation of SAs by the attack of oxidants at the central *N* atom.^10,17^

#### C–S Bond Cleavage

The cleavage of the C–S bond of SAs during hydrolysis to form aniline as a product has been reported. Similar to S–N bond cleavage, the reaction is promoted in an acidic environment and at higher temperatures.^11^ Aniline was also formed by direct photolysis.^10,17^

As already mentioned, two SA drugs, namely, SMX and SMZ, were not cleaved at all. In contrast to all other SA tested here, SMX has a five-membered heterocycle that is known^11^ to be less reactive than six-membered toward aromatic nucleophilic substitution. The other effects that may hinder the reactivity of SA drugs are the substituents on the Heterocyclic ring. While SP has none and was the most susceptible to hydrolysis followed by SDM with two methoxy groups, the hydrophobic methyl groups (one in SM and two in SMZ) seem to have a detrimental effect on the reactivity. Finally, the role of the leaving groups and their stabilization also play a major role in the nucleophilic sulfonyl substitution.

## Conclusions

Three Nanoceria samples with different physicochemical and particularly surface properties were used for the adsorption and chemical transformation of several sulfonamide antibiotics, namely, sulfadimethoxine (SDM), sulfamerazine (SM), sulfapyridine (SP), sulfomethoxazole (SMX), and sulfamethazine (SMZ). Interestingly, the spontaneous cleavage of SDM, SM, and SP on the surface of all three Nanoceria samples without any illumination, pH adjustment, or any other activation was demonstrated and described for the first time. Three independent methods, HPLC-DAD, LC-MS/MS, and NMR spectroscopy, were used to identify the reaction products and monitor the reaction kinetics. The products identified as sulfanilic acid, sulfanilamide, and aniline are typical products that arise from acid hydrolysis of SAs, indicating that ceria-catalyzed hydrolysis is the likely reaction mechanism.

While cleavage of the S–N bond giving sulfanilic acid was the most common mechanism, breaking of C–N and C–S bonds was also observed, yielding sulfanilamide and aniline, respectively, but mainly on the CePER sample that contained the most surface defects and surface OH groups. The formation of different products was probably related to the variability and strength of possible active sites on the surface of different ceria samples as well as to the susceptibility of individual bonds in SA drugs to their cleavage. It should be mentioned that the more resistant sulfonamides, SMX and SMZ, were neither adsorbed nor spontaneously cleaved by CeO_2_.

Nevertheless, based on the results presented here, we postulate that CeO_2_ reactivity is not limited to well-known dephosphorylation reactions but can be relevant to substances containing sulfonamide and sulfonyl (and similar) functional groups.

## Supporting Information Available

Synthesis methods, detailed degradation procedure, HPLC and XPS measurement details, additional TEM analysis data, nitrogen physisorption data, XPS analysis, calculated crystallite size of the nanoceria, ^1^H - ^1^H COSY NMR spectra and spectra of standards, LC-MS analysis data, and kinetics data for the degradation of SDM in ACN (PDF)
