# Supplementary material for: Ceria-Catalyzed Hydrolytic Cleavage of Sulfonamides
Source: Inorg Chem. 2024 Jan 18;63(4):2298–309. doi: 10.1021/acs.inorgchem.3c04367 (PMC10828983; doi:10.1021/acs.inorgchem.3c04367)
Supplement: Supplementary file 1 — ic3c04367_si_001.pdf [file ic3c04367_si_001.pdf]

SUPPORTING INFORMATION

Ceria-catalyzed hydrolytic cleavage of sulfonamides

Jiří Henych,[*†,‡] Martin Šťastný,[†] Sylvie Kříženecká,[‡] Jan Čundrle,[§] Jakub Tolasz,[†] Tereza Dušková,[§] Martin Kormunda,[§] Jakub Ederer,[‡] Štěpán Stehlík,[∥] Petr Ryšánek,[§] Viktorie Neubertová,[§] and Pavel Janoš[‡]*

† Institute of Inorganic Chemistry of the Czech Academy of Sciences, 250 68 Husinec-Řež, Czechia

‡ Faculty of Environment, Jan Evangelista Purkyně University in Ústí nad Labem, Pasteurova 3632/15, 400 96 Ústí nad Labem, Czechia

§ Faculty of Science, Jan Evangelista Purkyně University in Ústí nad Labem, Pasteurova 3632/15, 400 96 Ústí nad Labem, Czechia

|| Institute of Physics of the Czech Academy of Sciences, Cukrovarnická 10, 162 00 Prague, Czechia

*Corresponding author e-mail address: henych@iic.cas.cz

Sample synthesis details. The CeAMM sample: 1L of $Ce(NO_3)_3 \times 6H_2O$ aqueous solution (0.01 M) was pre-heated to 60 ˚C in a closed stirred glass reactor under constant bubbling with ambient air with CO_2 removed by bubbling the air first through KOH aqueous solution (1 M). After 30 min, the solution was precipitated with 20 mL of ammonium hydroxide (25%) solution. The mixture was stirred for another 4 hours until yellow precipitate was formed. The precipitate was rinsed with water by decantation and using dialysis membrane, then filtered and air-dried.

The CePER sample: 0.1 L of $Ce(NO_3)_3 \times 6H_2O$ aqueous solution (0.1 M) was precipitated with NaOH aqueous solution 1 M) to reach basic pH under constant stirring. The formed gelatinous mass was rinsed with water by decantation and 0.1 L of hydrogen peroxide (30%) was added while colling the mixture in an ice bath. The brown-orange suspension formed was left

aging for two hours and then refluxed at 100 ˚C for 24 hours. The yellowish precipitate was rinsed with water by decantation and using dialysis membrane, then filtered and air-dried.

The CeUREA sample: 1L of $Ce(NO_3)_3 \times 6H_2O$ aqueous solution (0.02 M) was mixed with 200 g of urea and the solution was heated at 92 ˚C for 5 hour. The evaporated water was regularly replenished during the entire process. After cooling down, the precipitate was rinsed with water by decantation and using dialysis membrane, then filtered and air-dried. Subsequently, the powder was then calcined in a furnace for 2 hours at 500 ˚C with a temperature increase of 5 ˚C/min.

XPS measurement details. The samples were loaded into the XPS apparatus in dry powder form. Drying was completed in the load-lock chamber of the XPS instrument, where a pressure of about $4x10^{-7}$ mbar was reached before introducing the samples into the analytical chamber of the XPS instrument. An Al anode at pass energy 40 eV and 10 eV was used to obtain a survey spectrum and a high-resolution spectrum, respectively. The spectra were collected in the normal direction and the sample charge was not compensated during the measurement. The analyzer was set to operate in Fixed Analyzer Transmission mode and Medium Area setting (Magnification M

= 5) with an entrance slit of 7 × 20 mm^2 and an aperture diameter of 35 mm, so the measured area was about 1.4 × 4 mm^2. During the measurement, the pressure was kept below 7 x 10^{-7} Pa. The acquired data were processed in CasaXPS software with a Shirley background profile and the built-in RSF was used to calculate the elemental composition. Samples were placed for carbon double-sided tape in sufficient quantities to completely cover the tape so that no carbon from the tape is detectable in the spectra. The pump down time was the minimum necessary and the load lock time was about 90 minutes.

The atomic concentration of oxygen was calculated from the high-resolution spectra of O1s and Ce3d (with using RSF factors in CasaXPS software), then the chemical bonding in the O1s high resolution spectra were analyzed and -OH groups identified. The oxygen peak was deconvoluted to bulk oxygen and -OH groups and the relative percentage of -OH in O1s spectra was multiplied by the atomic concentration of oxygen in the samples to obtain at.%.

HPLC-DAD measurement details. HPLC system was equipped with a high-pressure pump, column thermostat, autosampler and DAD detector. A Gemini® column, grain size 3 μm, stac. was used for the separation of SA drugs and their reaction products. phase C18, 110 Å

(manufactured by Phenomenex®, USA) with dimensions of 100 x 4.6 mm. The measurement was realized in the gradient elution mode, the mobile phase: acetonitrile (compartment C) with acetate buffer - sodium acetate solution (c = 0.02 M) with the addition of acetic acid adjusted to pH ~ 4.5 (compartment A). The flow rate of the mobile phase was 0.7 mL/min. The separation was realized at a temperature of 30 ˚C and the injection volume was 10 μl. The gradient elution settings for individual drugs and their degradation products are presented below (Table S1) together with the specific optimized conditions for the detection of drugs and their degradation products (Table S2).

Table S1. Gradient elution setup.

**Time**	**%A**	**%C**	**Flow rate, ml/min**
-2 min (equilibration)	95	5	0.7
0 min	95	5	0.7
20 min	40	60	0.7

Table S2. Optimized conditions for the analysis of individual drugs and their degradation products.

Analyte	Wavelength, nm	Retention time, min
Sulfanilic acid	250	1.82
Sulfanilamide	260	3.85
Aniline	250	8.63
Sulfapyridine	260	9.21
Sulfamerazine	270	9.70
Sulfadimethoxine	270	16.11

LC-MS/MS measurement details. Luna column, 2.1 µm, C18, 100 x 3 mm heated at 40 ˚C. Chromatographic separation was achieved using a binary mobile phase (water (A) with 0.5 mM NH_4F + 5 mM CH_3COONH_4/acetonitrile (B)) delivered in the gradient mode at a flow rate of 0.3 – 0.4 mL/min. Gradient elution started at 10% of B (0 – 6 min) and linearly increased to 100% of B 0.1 min; then maintained at 100% of B for 60 sec (6 – 7 min); then decreased to 10% of B (7.1 – 11 min) for re-equilibration of the column. The injection volume of the samples was 100 µl. Mass spectrometric detection was performed using an MRM and SIM scan with an electrospray ionization source in positive mode (ESI+). The measurement conditions: gas temperature 150 ˚C;

gas flow 15 L/min; ion spray voltage 3000 V; sheat gas temperature 400°C; sheat gas flow 11 L/min; nebulizer 30 psi; nozzle voltage 500 V.

NMR spectroscopy measurements details. The reaction mixtures of CeAMM, CePER and CeUREA samples with SDM were concentrated by vacuum manifold before the measurement. 0.5 ml of each sample was collected in an NMR cuvette containing a capillary sealed with deuterated solvent. ^{1}H and ^{1}H-^{1}H COSY experiments were used to determine the individual signals in samples. The chemical shifts, integration, coupling constant, distances and appearance of signals were compared with the signals of the expected compounds SDM and the decomposition products, i.e. aniline, sulfanilamide and sulfanilic acid.

Table S3. Crystallite sizes calculated using the Scherrer equation.

Sample			Crystallite size, Å		
	(111)	(200)	(220)	(311)	Average
CeAMM	53	46	48	40	47
CePER	68	46	39	33	47
CeUREA	117	121	102	102	111

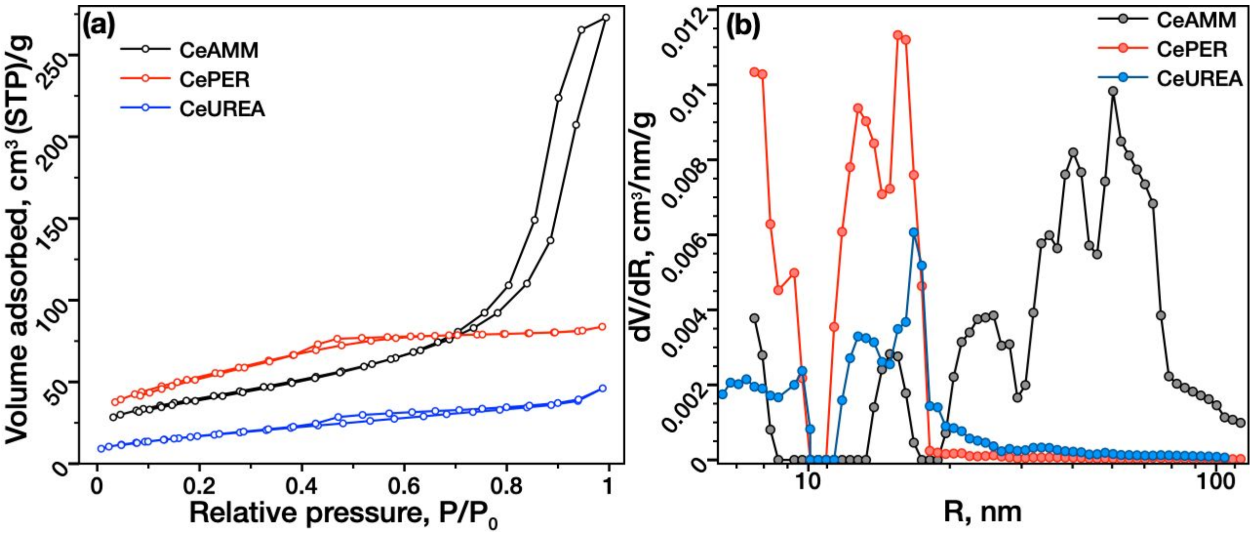

Figure S1. (a) Nitrogen sorption isotherms and (b) pore size distribution (DFT method) of prepared ceria samples.

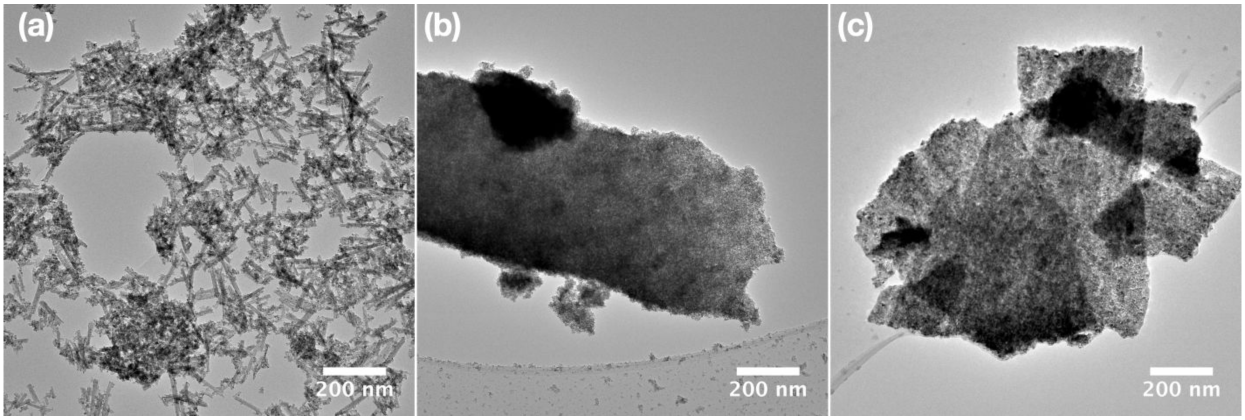

Figure S2. TEM analysis of (a) CeAMM, (b) CePER, and (c) CeUREA samples.

XPS analysis details. The survey spectra showed only cerium and oxygen related peaks. The Ce 3d was deconvoluted according to the previous work (https://doi.org/10.1016/j.apsusc.2009.02.080). The XPS spectrum of Ce 3d consists of three 3d3/2 - 3d5/2 spin-orbit-split doublets representing different $4f^0$ (Ce^{4+}) configurations in the photoemission final state, and two other doubles represent $4f^1$ (Ce^{3+}) states.

In more details, the highest binding energy peaks, u``` and v``` located at about 916.9 eV and 898.5 eV, respectively, are results of the Ce $3d^9$ $4f^0$ O $2p^6$ final state. The satellite peak u``` associated with the Ce 3d3/2 is characteristic of the presence of Ce^{4+}. The lowest binding energy states u, v, u``, and v``, respectively located at 901.1 eV, 882.7 eV, 907.3 eV, and 888.8 eV, are the results of the Ce $3d^9$ $4f^2$ O $2p^4$ and Ce $3d^9$ $4f^1$ O $2p^5$ final states. Ce^{3+} oxides have Ce 3d3/2

and Ce 3d5/2 spectra composed of two multiplets. The highest binding energy peaks, u` and v` located at about 903.4 eV and 885.0 eV, respectively, are the results of the Ce $3d^9$ $4f^1$ O $2p^6$ final state. The lower binding energy states, u^0 and v^0 respectively located at 899.6 eV and 881.1 eV, are the result of the Ce $3d^9$ $4f^2$ O $2p^5$ final state.

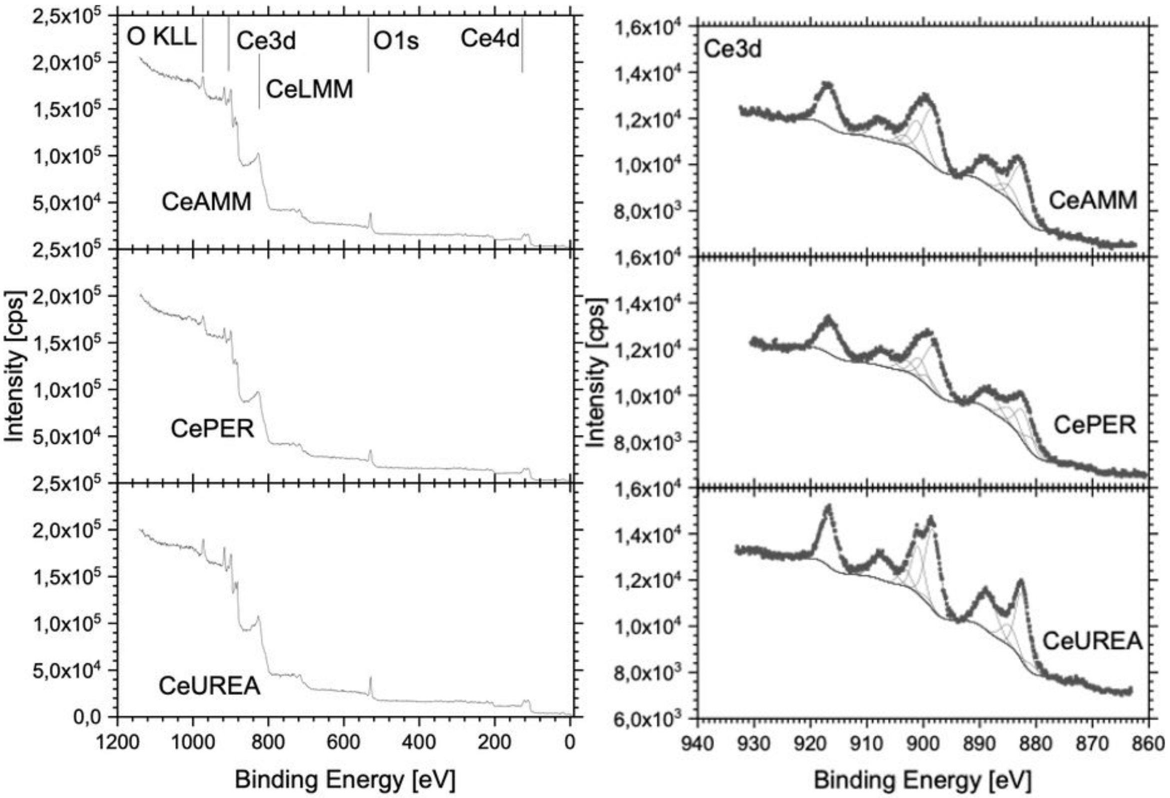

Figure S3. XPS (left) survey spectrum and (right) high-resolution Ce 3d spectrum of prepared samples.

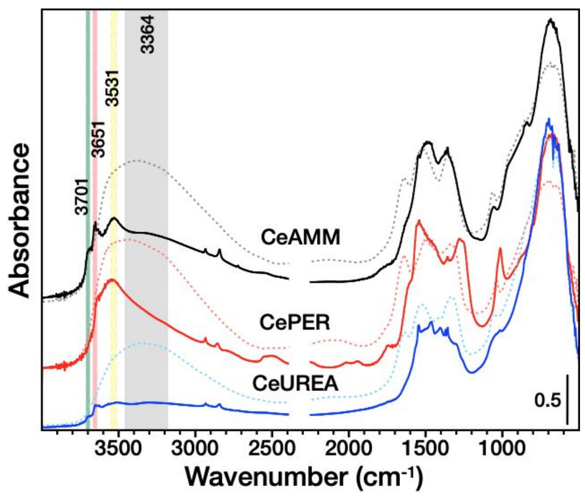

Figure S4. In situ DRIFT spectra of the ceria samples recorded at RT (dotted lines) and 200 ˚C (solid lines) under the flow of nitrogen.

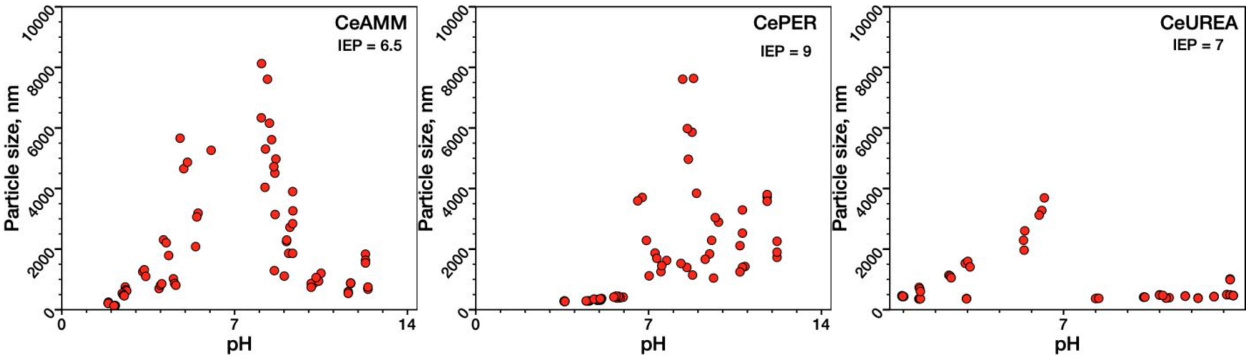

Figure S5. Hydrodynamic particle size as a function of pH measured by DLS.

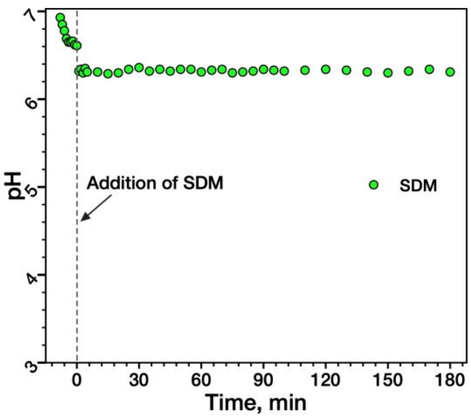

Figure S6. The pH measurement of the solution of SDM (10 mg/L) in RO water.

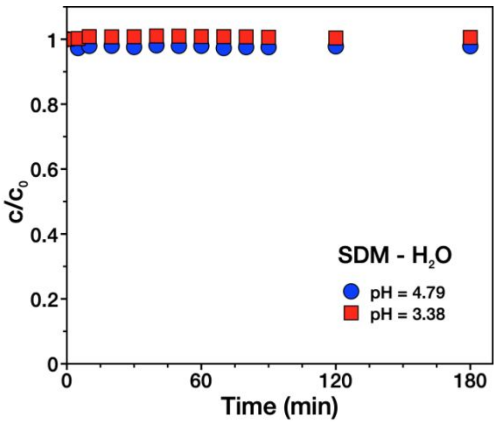

Figure S7. The kinetics of SDM degradation at pH 3.38 and 4.79 without ceria catalyst.

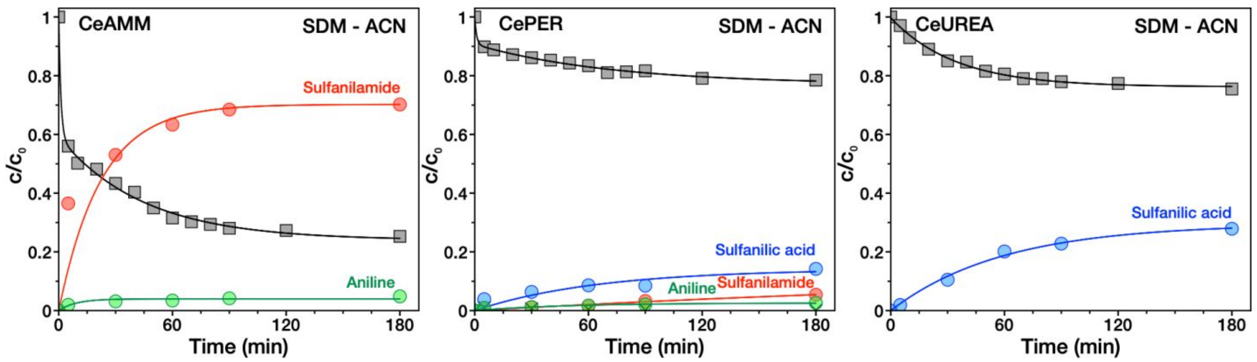

Figure S8. The kinetics of adsorption and degradation of SDM on nanoceria samples in acetonitrile (ACN).

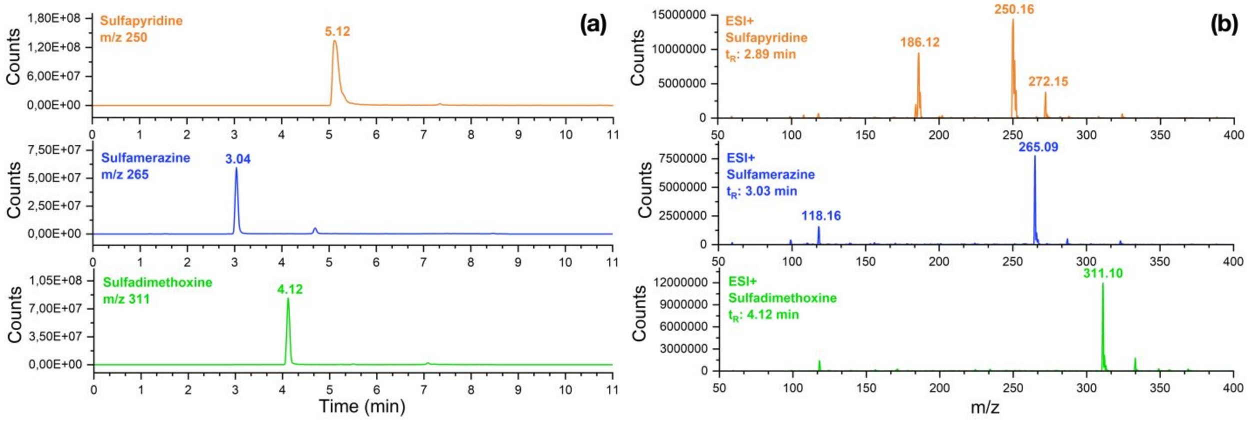

Figure S9. LC-MS extracted ion chromatograms of reference standards (SDM, SM, SP) and corresponding MS-ESI+ spectra at the respective retention times.

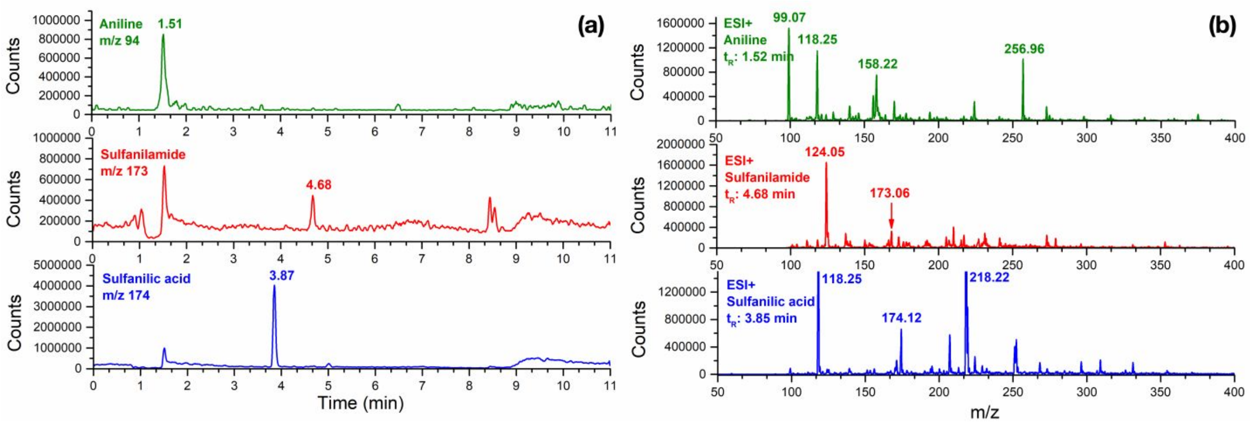

Figure S10. (a) LC-MS extracted ion chromatograms of reference standards of the expected degradation products (sulfanilic acid, sulfanilamide, aniline) and (b) corresponding MS-ESI+ spectra at the respective retention times.

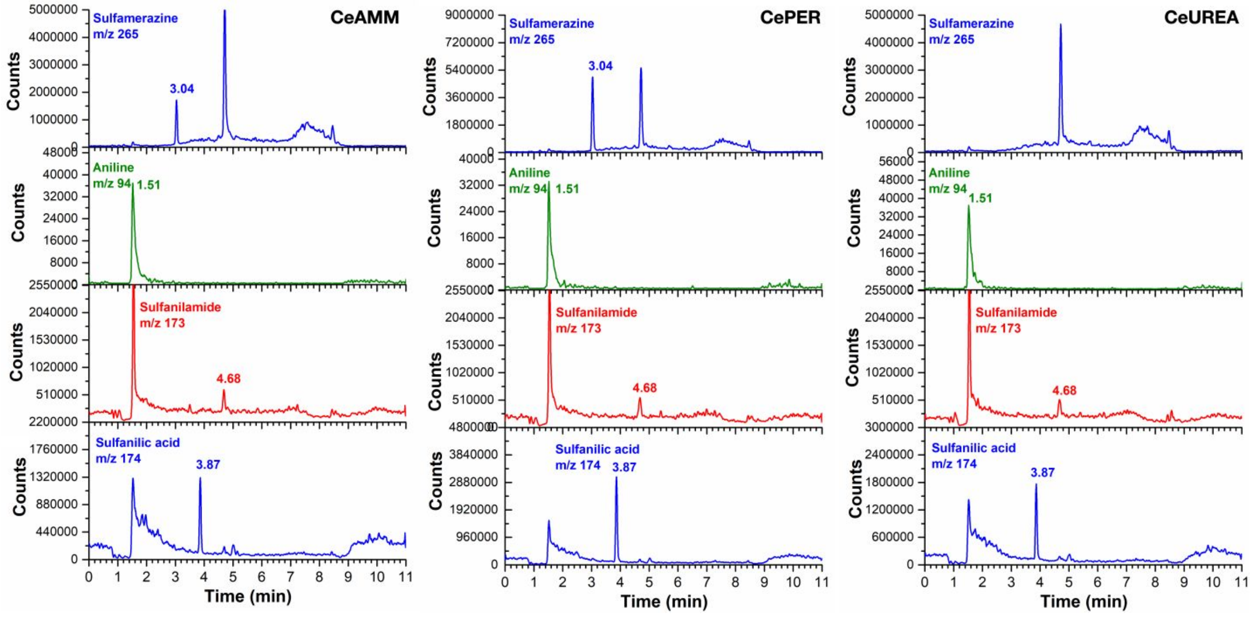

Figure S11. LC-MS analysis of samples obtained by extraction of different ceria samples after 180 min reaction with SM.

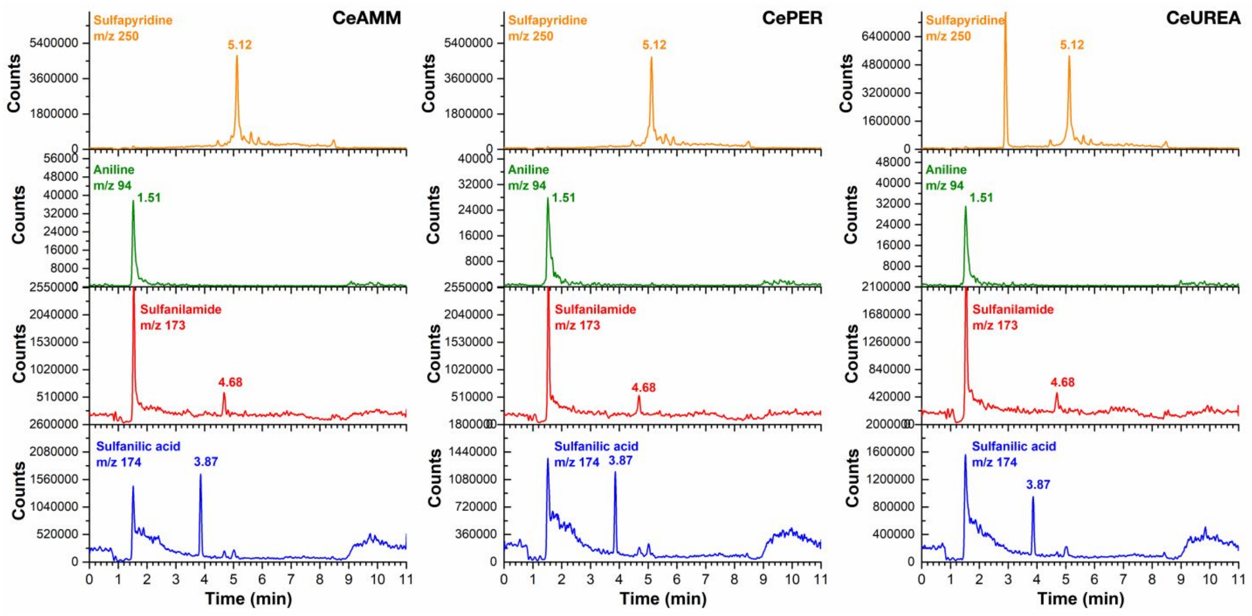

Figure S12. LC-MS analysis of samples obtained by extraction of different ceria samples after 180 min reaction with SP.

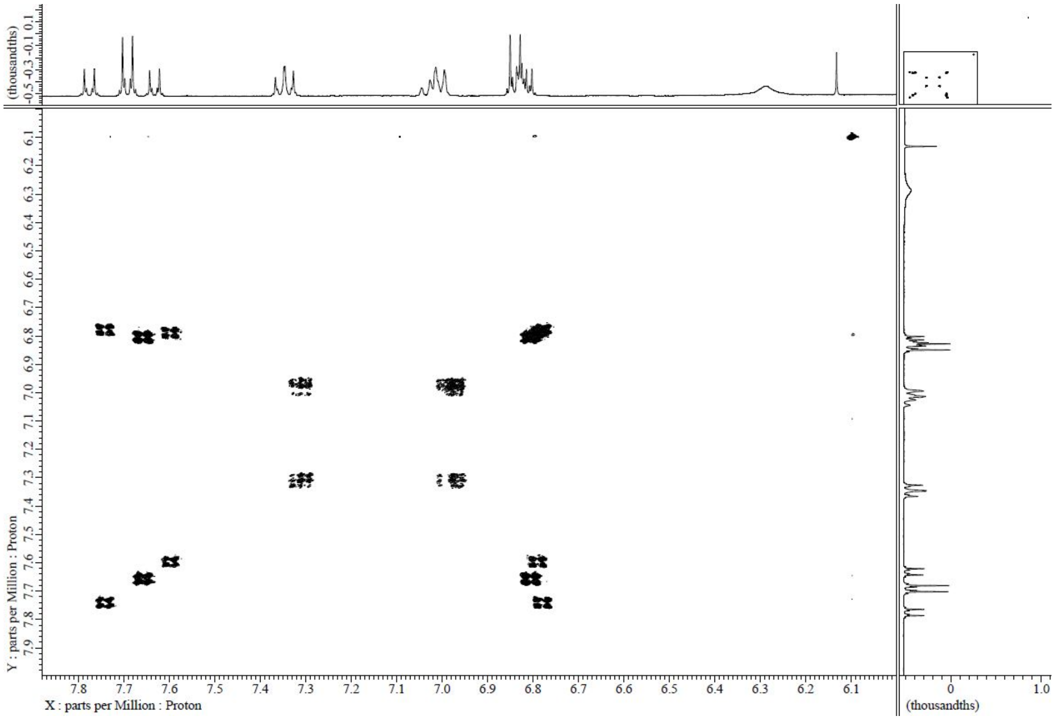

Figure S13. ^{1}H - ^{1}H COSY NMR spectrum of the sample CeAMM in DMSO-d_6

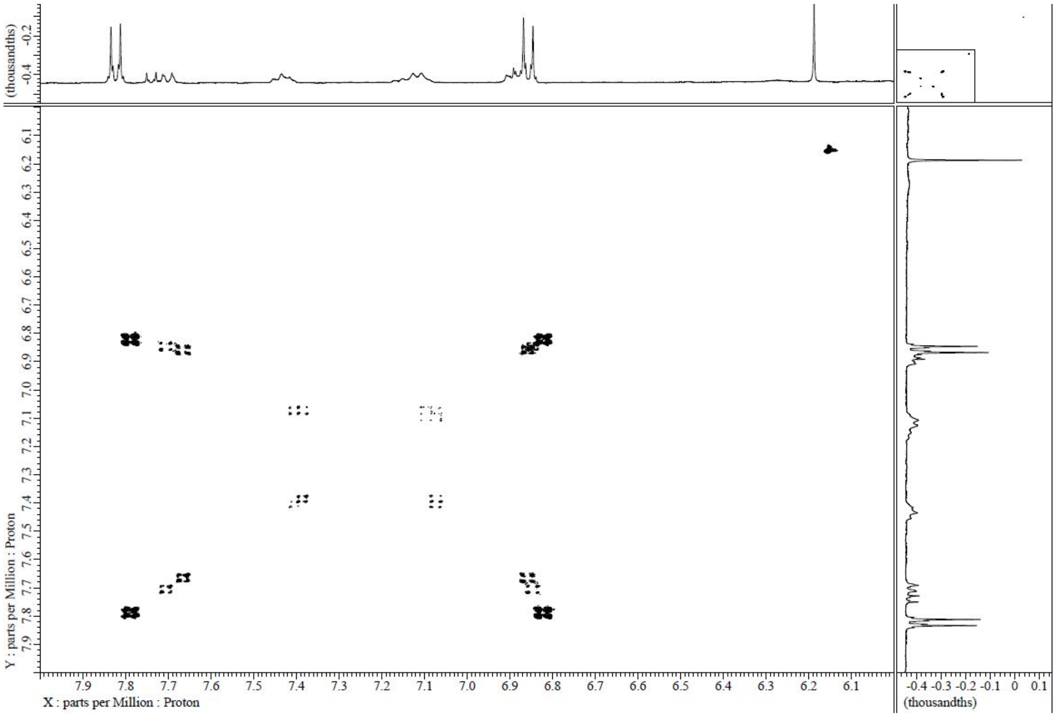

Figure S14. ^{1}H - ^{1}H COSY NMR spectrum of the sample CePER in DMSO-d_6

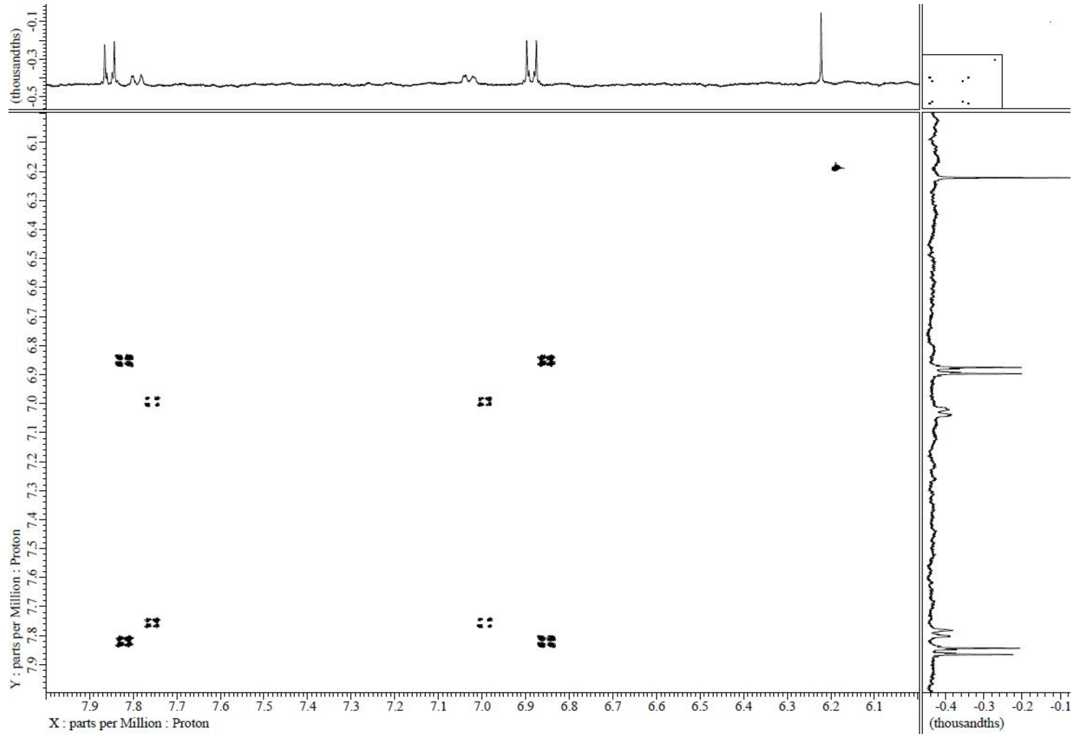

Figure S15. ^{1}H - ^{1}H COSY NMR spectrum of the sample CeUREA in DMSO-d_6

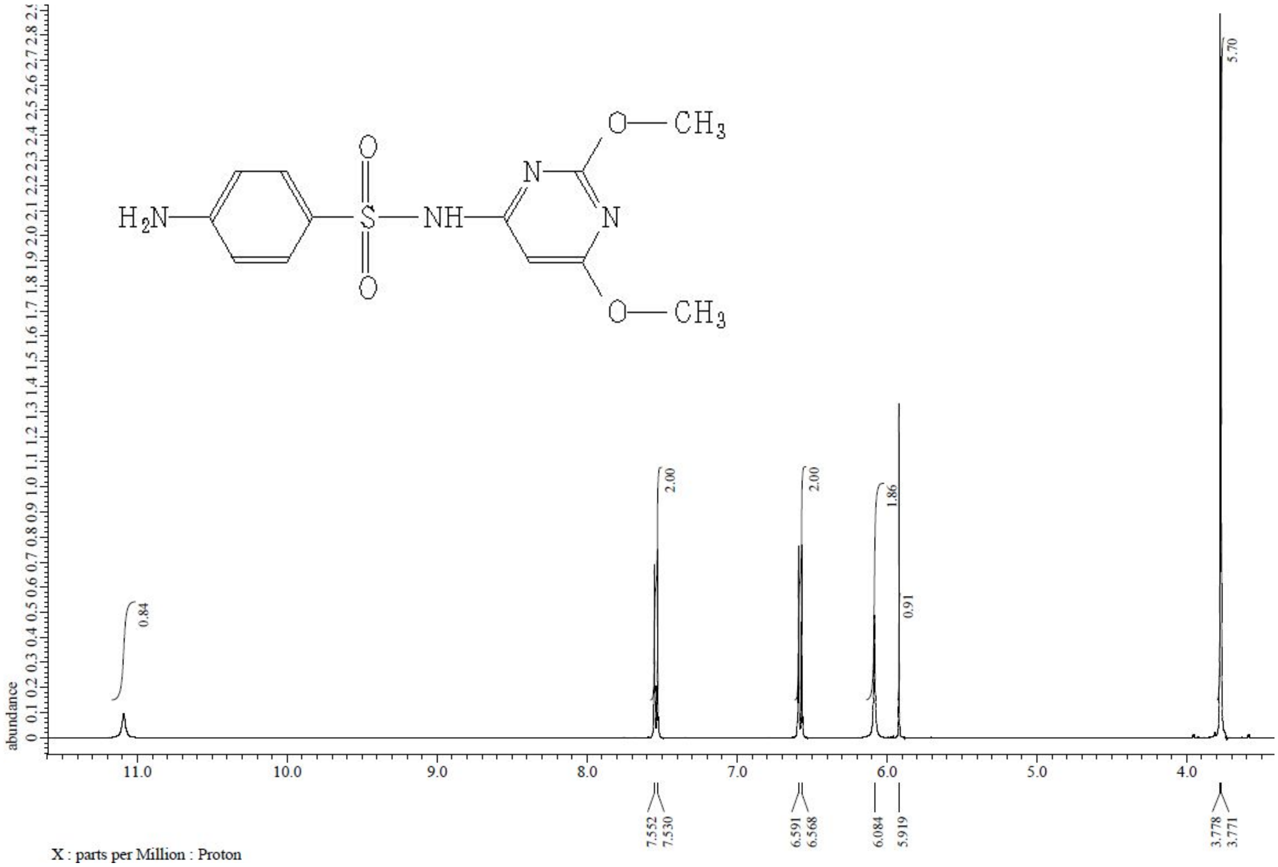

Figure S16. ^{1}H NMR spectrum of sulfadimethoxine in DMSO-d$_6$

NMR(DMSO-d$_6$): ^{1}H (399.78 MHz) δ 3.77, 3.78 (2 × s, 6H, OCH_3), 5.92 (s, 1H, *H*(pyrimidinyl)), 6.08 (br s, 2H, NH_2), 6.58 (app d, AA´XX´, ^3J = 8.9 Hz, 2H, 2 × C*H*(sulfonamide)), 7.54 (app d, AA´XX´, ^3J = 8.7 Hz, 2H, 2 × C*H*(sulfonamide)), 11.10 (br s, 1H, N*H*)

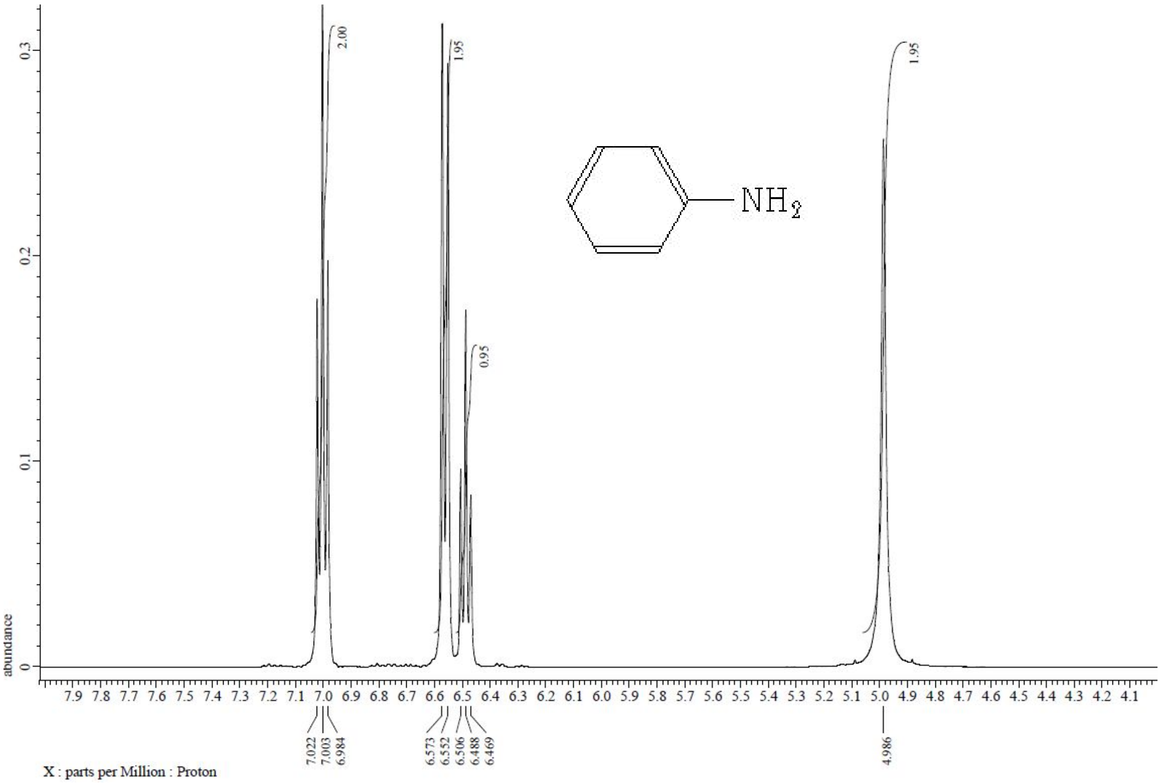

Figure S17. ^{1}H NMR spectrum of aniline in DMSO-d_6

NMR(DMSO-d_6): ^{1}H (399.78 MHz) δ 4.99 (br s, 2H, N*H$_2$*), 6.49 (app t, AA´MM´X, ^{3}J= 7 Hz, 1H, 1 × C*H*), 6.56 (app d, AA´MM´X, ^{3}J= 9.7 Hz, 2H, 2 × C*H*), 7.00 (app t, AA´MM´X, ^{3}J= 7 Hz, 2H, 2 × C*H*)

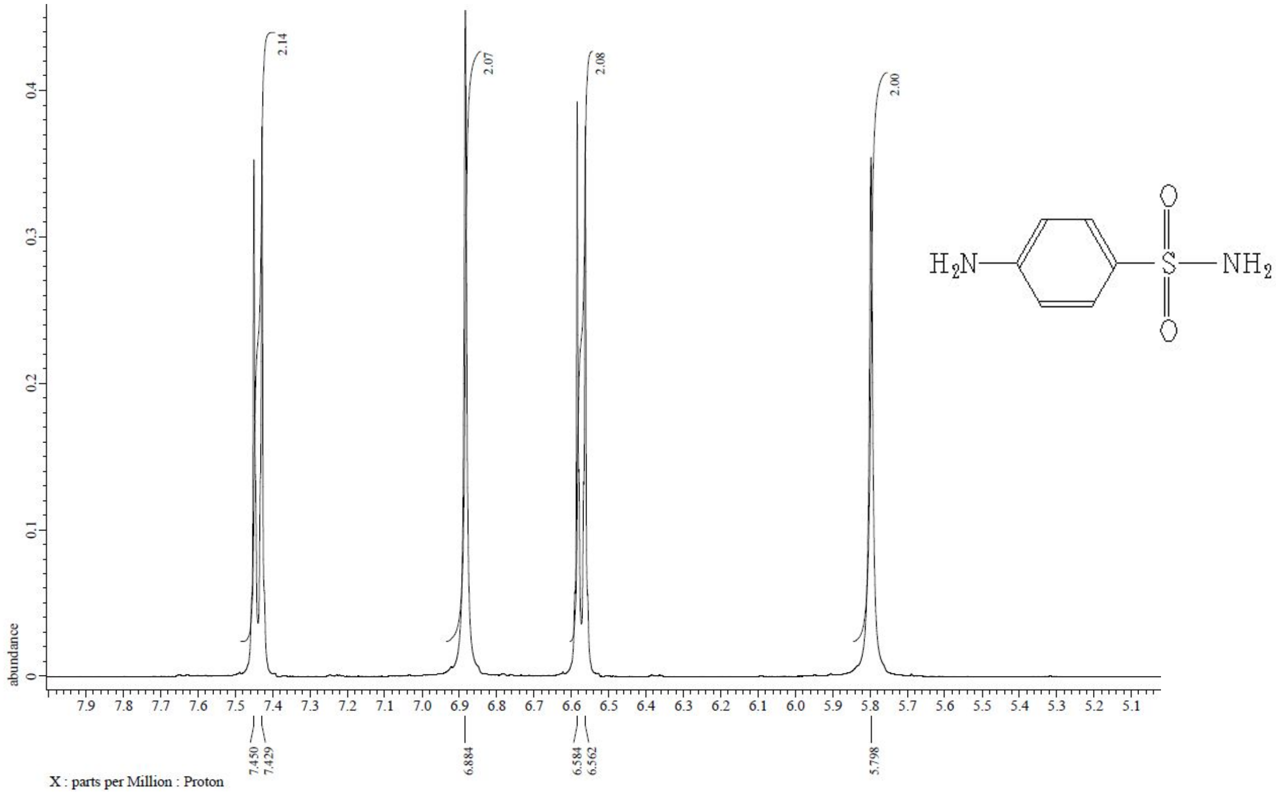

Figure S18. ^{1}H NMR spectrum of sulfanilamide in DMSO-d$_6$

NMR(DMSO-d$_6$): ^{1}H (399.78 MHz) δ 5.80 (br s, 2H, N*H_2*), 6.57 (app d, AA´XX´, ^3J= 8,7 Hz, 2H, 2 × C*H*), 6.88 (br s, 2H, N*H_2*) 7.44 (app d, AA´XX´, ^3J= 8,7 Hz, 2H, 2 × C*H*)

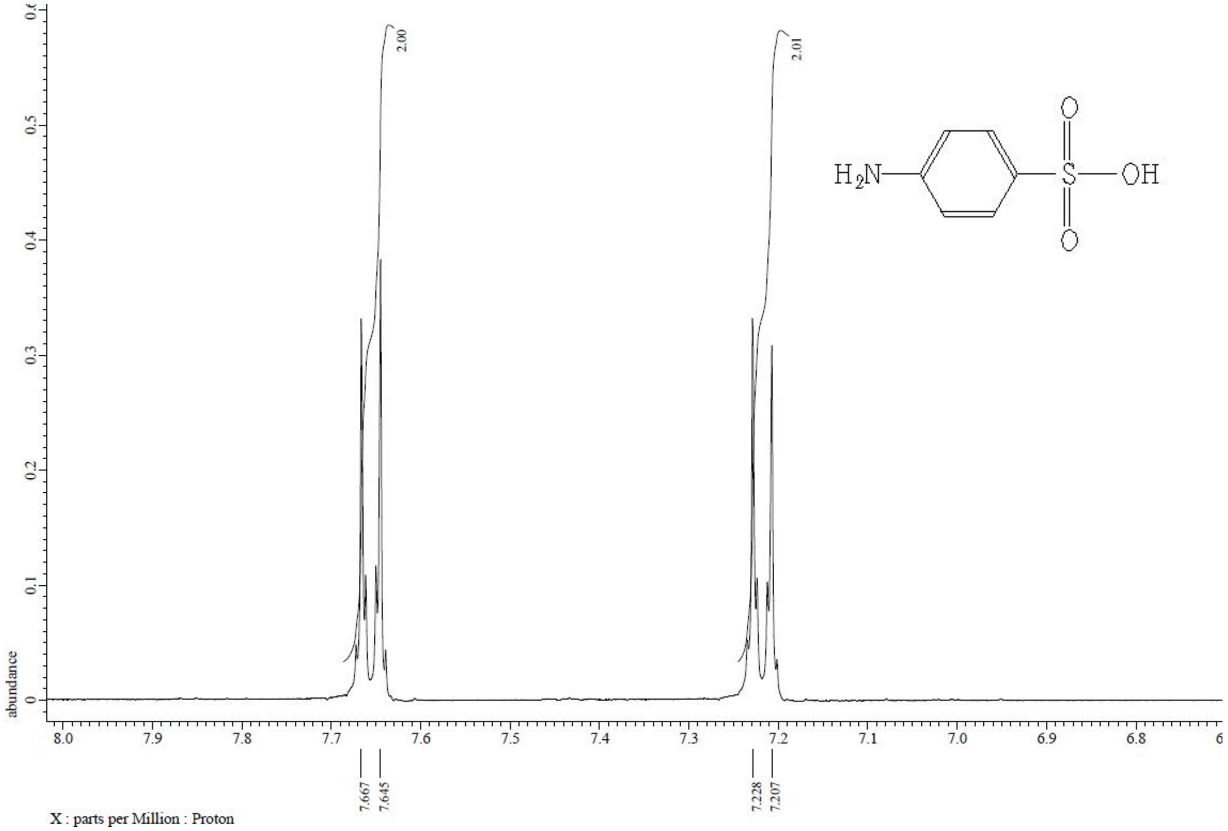

Figure S19. ^{1}H NMR spectrum of sulfanilic acid in DMSO-d_6

NMR(DMSO-d_6): ^{1}H (399.78 MHz) δ 7.23 (app d, AA´XX´, ^3J= 8,7 Hz, 2H, 2 × C*H*), 7.67 (app d, AA´XX´, ^3J= 8,7 Hz, 2H, 2 × C*H*)
